# Antibiotic-induced alterations and repopulation dynamics of yellowtail kingfish microbiota

**DOI:** 10.1186/s42523-020-00046-4

**Published:** 2020-08-03

**Authors:** Thibault P. R. A. Legrand, Sarah R. Catalano, Melissa L. Wos-Oxley, James W. Wynne, Laura S. Weyrich, Andrew P. A. Oxley

**Affiliations:** 1 School of Biological Sciences, The University of Adelaide, Adelaide, SA Australia; 2 CSIRO, Agriculture and Food, Hobart, TAS Australia; 3 South Australian Research and Development Institute, Aquatic Sciences Centre, West Beach, SA Australia; 4 College of Science and Engineering, Flinders University, Adelaide, SA Australia; 5 Department of Anthropology and Huck Institutes of Life Sciences, The Pennsylvania State University, University Park, PA USA; 6 Faculty of Science Engineering and Built Environment, School of Life and Environmental Sciences, Deakin University, Geelong, Victoria Australia

**Keywords:** 16SrRNA, Microbiome, FMT, Antibiotics, Fish

## Abstract

**Background:**

The use of antibiotics in aquaculture is a common infection treatment and is increasing in some sectors and jurisdictions. While antibiotic treatment can negatively shift gut bacterial communities, recovery and examination of these communities in fish of commercial importance is not well documented. Examining the impacts of antibiotics on farmed fish microbiota is fundamental for improving our understanding and management of healthy farmed fish. This work assessed yellowtail kingfish (*Seriola lalandi*) skin and gut bacterial communities after an oral antibiotic combination therapy in poor performing fish that displayed signs of enteritis over an 18-day period. In an attempt to promote improved bacterial re-establishment after antibiotic treatment, faecal microbiota transplantation (FMT) was also administered via gavage or in the surrounding seawater, and its affect was evaluated over 15 days post-delivery.

**Results:**

Antibiotic treatment greatly perturbed the global gut bacterial communities of poor-performing fish – an effect that lasted for up to 18 days post treatment. This perturbation was marked by a significant decrease in species diversity and evenness, as well as a concomitant increase in particular taxa like an uncultured *Mycoplasmataceae* sp., which persisted and dominated antibiotic-treated fish for the entire 18-day period. The skin-associated bacterial communities were also perturbed by the antibiotic treatment, notably within the first 3 days; however, this was unlike the gut, as skin microbiota appeared to shift towards a more ‘normal’ (though disparate) state after 5 days post antibiotic treatment. FMT was only able to modulate the impacts of antibiotics in some individuals for a short time period, as the magnitude of change varied substantially between individuals. Some fish maintained certain transplanted gut taxa (i.e. present in the FMT inoculum; namely various *Aliivibrio* related ASVs) at Day 2 post FMT, although these were lost by Day 8 post FMT.

**Conclusion:**

As we observed notable, prolonged perturbations induced by antibiotics on the gut bacterial assemblages, further work is required to better understand the processes/dynamics of their re-establishment following antibiotic exposure. In this regard, procedures like FMT represent a novel approach for promoting improved microbial recovery, although their efficacy and the factors that support their success requires further investigation.

## Introduction

Aquaculture is the fastest growing sector in the food animal industry [1]. However, its development is not without challenges. Due to the intensive methods used in production, diseases are common and often require the use of therapeutics. While there are a range of alternative treatment options available (e.g. probiotics, prebiotics, synbiotics, postbiotics, phytobiotics, phage therapy, or quorum sensing interference), antibiotics remains the most common therapy used in some aquaculture sectors to treat microbial infections [2, 3]. Worldwide, 67 antibiotic compounds were reported to be used in 11 of the top 15 highest producing countries, with oxytetracycline, sulphadiazine, and florfenicol the most commonly used in the industry [4]. In some countries, usage has increased substantially in recent years. For instance, antibiotic use doubled over a three-year period (from 143 to 382 tons between 2013 and 2016) in the Chilean salmon industry, which was largely attributed to the increased use of florfenicol and oxytetracycline for combatting *Piscirickettsia salmonis* infection [5]. While antibiotic use varies significantly between countries due to different laws and regulations [4, 6], there is widespread concern regarding the development of antimicrobial resistance in the global aquaculture industry and their broader impacts on the environment [5, 7–10].

The risks posed by the use of antimicrobials also include changes in an animal’s microbiota. Many of the antibiotics used in the aquaculture industry are considered to be broad-spectrum, and may indiscriminately act on both the pathogenic and commensal constituents [11]. Perturbation of the gut microbiota following antibiotic exposure has been reported in various fish species [8, 12, 13] and may be associated with changes in microbial enzymatic activity, gene expression, and protein and metabolite synthesis [14]. In humans and other animals, antibiotic use may have prolonged effects on the gut bacterial composition, leading to widespread perturbations and the extinction of some species [15]. Considering the importance of the microbiota in nutrient metabolism, digestion, and disease resistance [16], antibiotic-induced changes may be functionally detrimental, impacting the health and fitness of the animal. Knowledge of the specific impacts caused by select antibiotics as well as strategies that seek to minimise their effects on the fishes’ microbiota are thus likely to be a critical feature for supporting optimal performance and productivity of the system.

Prospects for overcoming or improving the inherent effects that antibiotics impose on the microbiota, or for optimising the overall health and fitness of fish in a production context, are increasing in demand and have been extensively studied within the last decade. This includes common strategies that aim to modulate the fish microbiota through the diet in order to improve disease resistance, nutrient digestibility, tolerance to stress, and reproduction [17]. More recently, however, procedures such as faecal microbiota transplantation (FMT) have be touted as a prospective, more holistic approach that has the capacity to improve outcomes by modulating the entire microbial community and facilitating the re-establishment of defunct species [18, 19]. First developed in 1958 to cure pseudomembranous enterocolitis in humans [20], FMT has since been used to successfully treat a range of other conditions including, among others, *Clostridioides difficile* infection, inflammatory bowel disease (IBD), and obesity [21]. Its role in mitigating the effects of antibiotics has also been recently demonstrated in humans and mice and has been shown to be more effective than treatment with probiotics, which instead resulted in a delayed or incomplete reconstitution of the microbiota [22]. In animal production systems, similar findings have also been reported for chickens, alongside improvements in nutritional capacity [23]. To the best of our knowledge, FMT has thus far not been investigated in fish in response to antibiotic-induced microbiota alterations, although experiments in African turquoise killifish (*Nothobranchius furzeri*) have demonstrated the power of the approach, revealing its capacity to restore bacterial diversity in old fish and influence longevity [24].

In Australia, the commercial production of valuable species, such as yellowtail kingfish (*Seriola lalandi*), is impeded by a variety of diseases including fluke infestation and gut enteritis [25, 26]. The latter is known to occur when fish are farmed at suboptimal temperatures and fed with a high proportion of soybean meal, although the mechanisms underlying this disease remain poorly understood and have limited treatment options available beyond antibiotics [26]. Such conditions have also been reported to be accompanied by changes in the bacterial diversity of the outer mucosa (skin and gills), suggesting a body-wide response [27]. An improved understanding of the effects of treatments, as well as new strategies that ameliorate treatment effects on the microbiota of fish suffering from gut disease, are warranted. Here, we investigated the influence of a novel antibiotic combination-therapy formulated for broad spectrum activity against a range of microorganisms (comprising commonly used oxytetracycline, as well as erythromycin and metronidazole) on the gut and skin mucosal microbiota of poor performing yellowtail kingfish (i.e. those suffering from enteritis); and the prospective role of FMT in gut microbiota repopulation.

## Methods

### Study design and experimental set-up

To assess the impacts of antibiotics and FMT on the gut and skin microbiota of yellowtail kingfish exhibiting symptomatic features of gut disease (as characterised by low body condition and weight loss), a total of 217 fish with a mean weight of ~ 1.6 kg were obtained from a single seacage (comprising fish of the same cohort, though of mixed genetics) under the auspices of a commercial aquaculture enterprise from temperate waters of southern Australia according to industry best practice veterinary care. Of these, 10 fish were randomly sampled to provide baseline bacterial community composition data. Fish were transported in a water tanker (with oxygen supplementation) to a research facility and housed in 5000 L tanks. Tanks were supplied with seawater at ambient temperature (12.7–14.0 °C; see Additional file 1: Table S1) from a flow-through system with mechanical filtration (drum filter). Additional water parameters were also recorded during the length of the experiment such as dissolved oxygen (94–115% saturation; see Additional file 1: Table S2), pH (7.59–7.73; see Additional file 1: Table S3), salinity (36–37‰; see Additional file 1: Table S4), ammonia concentration (< 0.25 ppm; see Additional file 1: Table S5), and CO_2_ concentration (always below detection level). Fish were fed to satiation once a day with the same proprietary feed formulation used in the commercial operation and were allowed to acclimatise for 3 weeks prior to the investigation. Tanks were flushed once a day to eliminate faeces at the bottom of the tanks. During acclimation, 15 fish were randomly sampled for histopathological examination to confirm their condition, revealing mild enteritis (as conducted by an external fish pathologist). After acclimation (~3 weeks), a total of 144 fish were distributed among 12 tanks (*n* = 12 fish/tank). The lengths and weights of all fish were recorded following brief sedation in 14 mg/L AQUI-S (AQUI-S New Zealand Ltd.) solution in surrounding seawater as described previously [26]. The total fish weight for each tank was recorded with an attempt to make these as even as possible (see Additional file 1: Table S6).

**Fig. 1:**
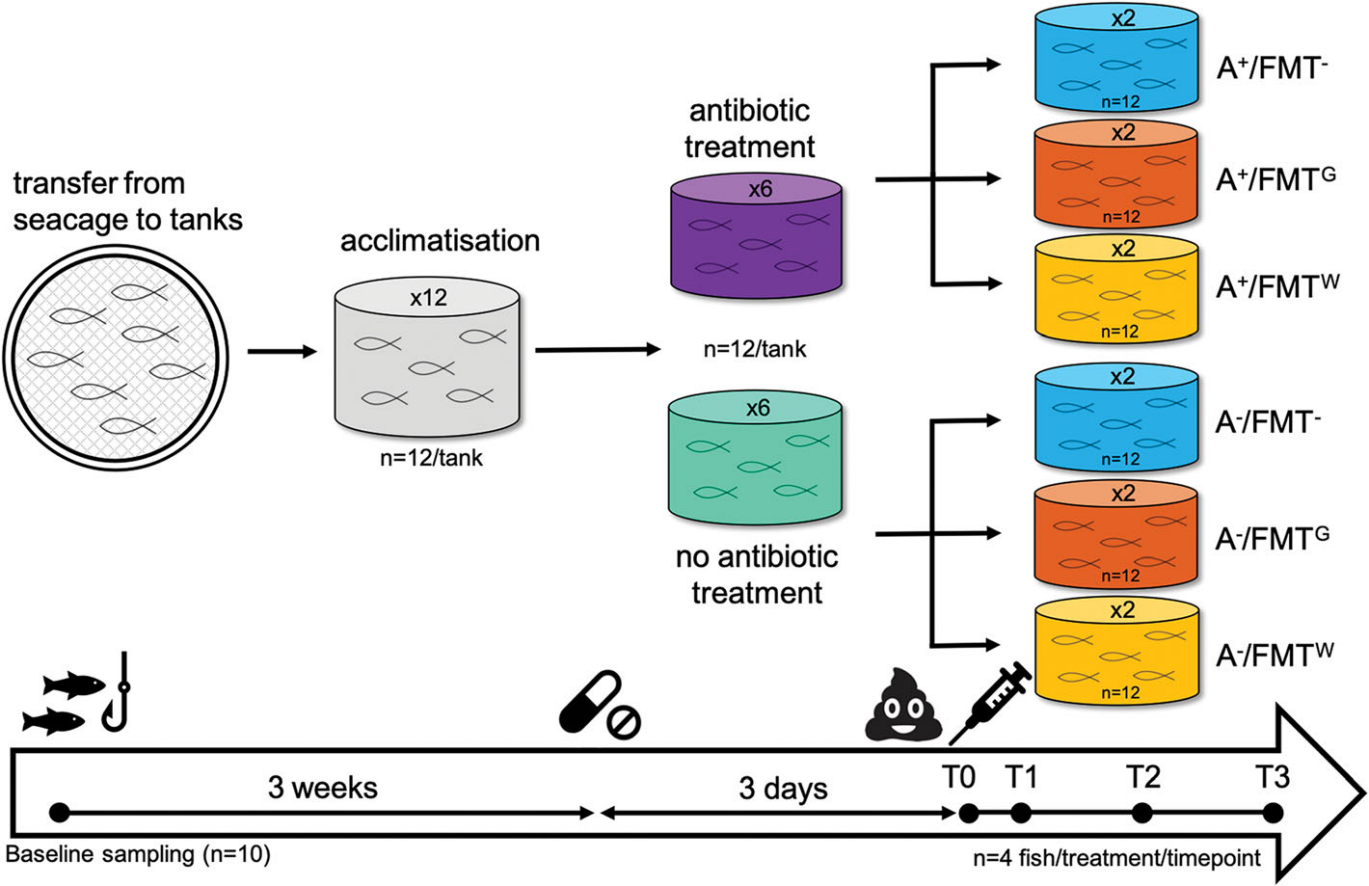
Overview of the experimental design of the study. A total of 6 treatment groups were compared and included no antibiotic treatment with no faecal microbiota transplantation (A^−^/FMT^−^); antibiotic treatment with no FMT (A^+^/FMT^−^); no antibiotic treatment with FMT via gavage (A^−^/FMT^G^); antibiotic treatment with FMT via gavage (A^+^/FMT^G^); no antibiotic treatment with FMT via water (A^−^/FMT^W^); and antibiotic treatment with FMT via water (A^+^/FMT^W^). Each treatment was replicated across 2 tanks. Antibiotic treatment was administered by oral gavage 3 days prior to FMT, with sampling conducted at: T0 = immediately prior to FMT; T1 = 2 days post FMT; T2 = 8 days post FMT; and T3 = 15 days post FMT

The experimental design comprised 6 treatment groups: (1) no antibiotic treatment with no FMT (A^−^/FMT^−^); (2) antibiotic treatment with no FMT (A^+^/FMT^−^); (3) no antibiotic treatment with FMT via oral gavage (A^−^/FMT^G^); (4) antibiotic treatment with FMT via oral gavage (A^+^/FMT^G^); (5) no antibiotic treatment with FMT via water (A^−^/FMT^W^); and (6) antibiotic treatment with FMT via water (A^+^/FMT^W^). Each treatment was replicated across two tanks of 12 fish (Fig. 1). Antibiotic treatment was administered by oral gavage 3 days prior to FMT treatment and consisted of a combination therapy comprising oxytetracycline (200 mg/kg), erythromycin (50 mg/kg) and metronidazole (50 mg/kg) (Sigma-Aldrich), which was prepared the morning of administration in polypropylene glycol (Sigma-Aldrich). Dose was determined in consultation with veterinary staff based on existing knowledge from Seriola or other species and was formulated to maximise the depletion of various types of gram positive and negative bacteria.

The FMT inoculum comprised the gut contents from 102 x ~ 3.5–4.5 kg healthy fish from a “healthy” seacage, where fish showed no signs of disease. The gut contents were obtained by the manual stripping of fish on-site at the commercial operation, which was immediately transported back to the research facility on ice for use in the trial on the same day. A total of 110 mL of feacal material was obtained and was made up to 400 mL in filter-sterilised seawater (as prepared using a 0.22 μm Nalgene™ Rapid-Flow™ filter unit, Thermo Fisher Scientific), and mixed thoroughly by vortexing. The inoculum was subsequently split into two parts, where one part (200 mL) was inoculated with one capsule (containing ~ 10 billion cells, ~ 5 × 10^7^ cells/mL) of the commercial probiotic *Lactobacillus rhamnosus* GG (LGG®) (Inner Health), a strain previously reported to have protective effects against pathogen infection [28], while the other part was left untreated; these are herein referred to as ‘spiked’ and ‘unspiked’ inoculum respectively. FMT was administered by oral gavage or in the surrounding seawater of the tanks 3 days post-antibiotic treatment, as described below.

For the treatment groups that received antibiotics and/or FMT via gavage, fish were first sedated in seawater comprising 14 mg/L AQUI-S (as described above) and then administered the treatment via a sterile 5 mL syringe fitted with a soft silicone tube [ø 5 mm] (Gecko Optical Scientific Equipment, Australia) that was just long enough to enter the stomach (~ 17 cm), as guided through a larger [ø 10 mm] flexible PVC tube. Care was taken to minimise stress by placing the fish on a cushioned surface, covering with a seawater saturated cloth, and gently restraining the fish during the brief procedure. A total of 1.5 mL of antibiotics and/or 3 mL of FMT inoculum was administered to each fish during the respective treatments.

For the treatment groups that received the FMT inoculum within the surrounding seawater, the water level of the tanks was dropped to ~ 1500 L. The tanks were cleaned to remove any accumulated faecal material and then 60 mL of the spiked or unspiked inoculum sample was added to a 5 liter bucket of seawater which was then added to the respective treatment tanks. Fish were then allowed to bath in the FMT inoculum treated water with no exchange (though with oxygen supplementation) for 3 hours before being refilled to full capacity.

### Sampling of fish

Alongside the 10 fish collected for baseline analyses, four fish per treatment/time point (*n* = 2 fish/replicate tank) were sampled over an 18 day period (i.e. at T0 [3 days post antibiotic treatment]; T1 [5 days post antibiotic treatment and 2 days after FMT]; T2 [11 days post antibiotic treatment and 8 days after FMT]; and T3 [18 days post antibiotic treatment and 15 days after FMT]) (Fig. 1). From each fish, a swab of the skin and a scraping of the hindgut was collected. Swabs of the skin were conducted as detailed earlier using sterile FLOQSwabs® (COPAN) [27]. For the hindgut, the gastrointestinal tract was first removed, separated from the fore and midgut, an incision made along its length with a sterile scalpel blade to expose the inner mucosal surface, and a scraping of the entire region obtained using a sterile glass microscope slide (with care taken to avoid excessive faecal material). Samples were stabilised immediately in RNAlater™ (Ambion) and stored at − 20 °C until downstream RNA extraction. In addition, 500 μL aliquots of the spiked and unspiked inoculum sample were placed in 15 mL tubes with 1.5 mL of RNAlater™ and stored at − 20 °C until downstream nucleic acid extraction. Finally, 1 liter of seawater was also taken from the seacage at the time of the initial fish collection, and 1 liter from a tank at the start and end of the experiment (i.e. at T0 and T3).

### Nucleic acid extraction, library preparation and Illumina sequencing

To investigate the active and thus likely resident bacterial community constituents, RNA was extracted from hindgut scrapings, skin swabs and spiked and unspiked FMT inoculum as described previously [27]. In brief, samples were placed into lysing matrix B tubes (MP Biomedicals) containing 1 mL of ice-cold RLT buffer supplemented with 1% β-mercaptoethanol v/v (Sigma-Aldrich). Bead-beating was performed to disrupt the samples using the FastPrep-24™ 5G instrument (MP Biomedicals) at an intensity setting of 5.5 for 45 s. Samples were then placed on ice, disrupted a second time using the same settings, and centrifuged at 14,000×g for 10 min at 4 °C. RNA was then extracted from the supernatant using the RNeasy mini kit (Qiagen) following the manufacturer’s instructions. The Turbo DNA-free™ kit (Life Technologies) was used to remove any contaminating gDNA. RNA extracts were then converted to cDNA using the Superscript™ III First Strand Synthesis System (Life Technologies) according to the manufacturer’s instructions.

To evaluate the contribution of the surrounding environmental bacterial consortia on the fish microbiota, DNA was extracted from the seawater samples following filtration onto 0.22 μM Nalgene™ Rapid-Flow™ filters (Thermo Fisher Scientific) using the FastDNA™ Spin Kit for Soil (MP Biomedicals) according to the manufacturer’s instructions. In addition, DNA was also extracted from the spiked and unspiked FMT inoculum samples using the same kit to evaluate the contribution of any taxa not represented in the RNA extracts. All samples were subsequently concentrated by ethanol precipitation using standard procedures, quantified using the NanoDrop 2000 spectrophotometer (ThermoFisher Scientific) and stored at − 20 °C prior to downstream library preparation.

The V1-V2 region of the 16S rRNA gene was amplified from the extracted cDNA and DNA extracts using universal eubacterial primers 27F and 338R, as described previously [27], and in conjunction with positive and negative (no template) PCR reagent controls. Briefly, two μL of cDNA and five μL of each sample were first subjected to 20 cycles of PCR, whereby one μL of this mixture from the first round was used as template in a further 15 cycles of PCR for incorporating individual barcodes and Illumina specific adaptors. Finally, one μL of the resultant mixture was used as a template in a final 10-cycle PCR for incorporating the Illumina multiplexing sequencing and index primers. Libraries were then purified using Agencourt AMPure XP beads (Beckman Coulter) and quantified using the Quant-iT™ Picogreen® dsDNA kit (Life Technologies) before being pooled in equimolar ratios and sequenced on the MiSeq platform (Illumina, San Diego, CA, United States) using 250 nt paired-end sequencing chemistry through the Australian Genome Research Facility (AGRF, North Melbourne, VIC, Australia).

### Bioinformatics and statistical analysis

Sequence reads were paired using PEAR (v0.9.5) where adapter sequences were also removed [29]. The merged fastq files were then processed and analysed using the QIIME2 (v2019.1) pipeline [30]. Demultiplexed paired-end sequence reads were truncated to a length of 300 bp, quality filtered and denoised into amplicon sequence variants (ASVs) using the DADA2 plugin [31]. Sequencing resulted in a total of 15,187,504 demultiplexed paired-end reads from 214 samples (average of 65,463 reads/sample, range 7254 – 159,600). Subsequent denoising, removal of reads associated with mitochondria, filtering of Eukaryote and unclassified Kingdom (after assigning taxonomy) sequences, and removal of samples with low coverage (< 9848 reads), resulted in 12,116,464 reads across 211 samples for downstream analysis. Each sample was rarefied to a depth of 9848 reads resulting in a total of 8255 ASVs in the dataset. Alpha rarefaction revealed sufficient sequencing coverage of the remaining samples (Additional file 2: Figure S1). Taxonomy was assigned to each ASV using the q2-feature-classifier against the Silva 132 99% OTUs reference sequences resource [32]. Alpha-diversity metrics (Shannon’s diversity, Pielou’s evenness, Faith’s phylogenetic diversity and total observed ASVs as a measure of richness) were estimated using q2-diversity. QIIME artefacts were imported into R using the package Qiime2R and plots were made using Phyloseq and ggplot2 [33]. Beta diversity metrics (Bray-Curtis dissimilarity matrix) and Principle Coordinate Analysis (PCoA) using the Bray-Curtis matrix were performed with Phyloseq. To investigate the influence of antibiotic and FMT treatment on the bacterial assemblages, read abundances for each ASV in the feature table were square-root transformed to down-weight the impact of a few extremely dominant ASVs. Statistical differences for the univariate measures, such as, alpha diversity were performed using 2-way ANOVA, accounting for both the treatment (i.e. antibiotic or FMT treatment) and time. For multivariate measures, significant differences between a priori predefined groups of samples were evaluated using both two-way and one-way permutational multivariate analysis of variance (PERMANOVA), allowing for type III (partial) sums of squares, fixed effects of sum to 0 for mixed terms, and exact *p*-values generated using unrestricted permutation of raw data [34], using the Adonis function in R. The function *pairwise.adonis* with Bonferroni correction was used to investigate the significance between timepoints when time was a significant factor from the PERMANOVA analysis. Differential abundance was assessed using Deseq2 with p-value corrected using the default Benjamini-Hochberg false discovery rate method, as suggested recently for the analysis of microbiome data with a small number of replicates per treatment (< 20) [35, 36]. In some cases (e.g. for some ASVs), the data distribution was assumed to not follow a normal distribution, so the non-parametric version of 2-sample or *k*-sample tests were performed (e.g. the Mann-Whitney U test or Kruskal-Wallis H test). In order to quantify the change in magnitude in the bacterial communities after antibiotic and FMT treatment, pair-wise comparisons between each pair of samples were made using the Bray-Curtis similarity algorithm, where a higher value indicates that samples share more ASVs of a similar abundance.

## Results

### Impact of acclimatisation on the gut and skin microbiota

**Fig. 2:**
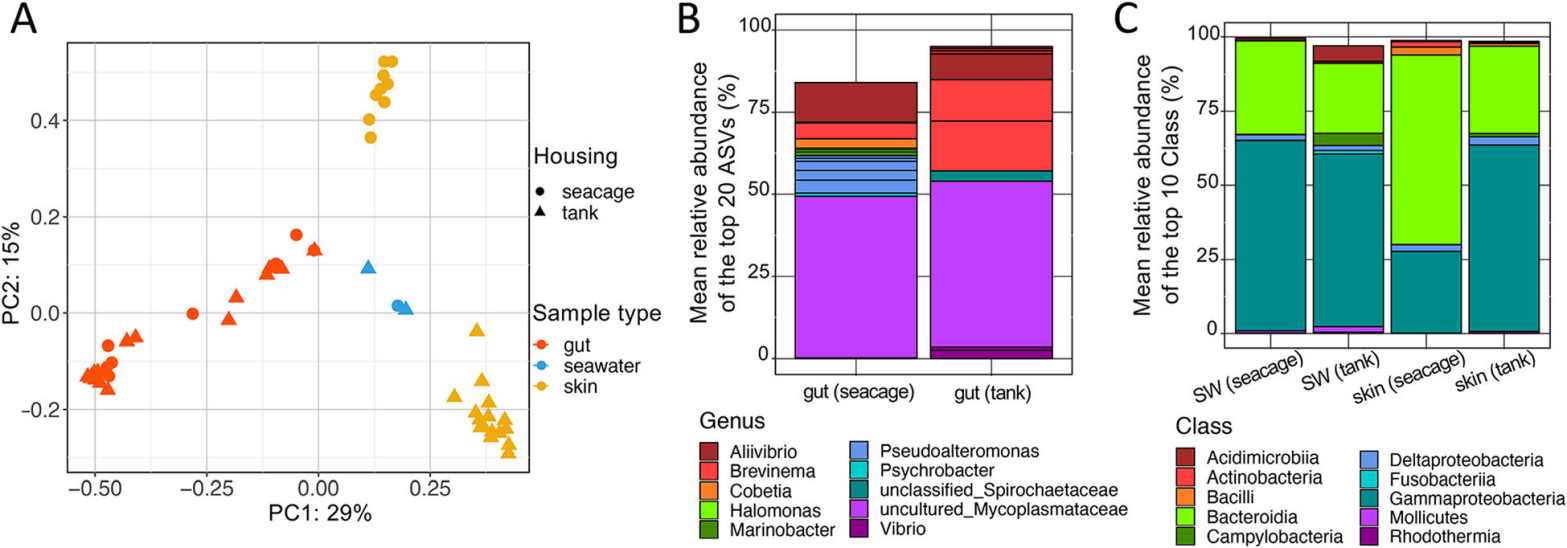
Impact of acclimatisation on the gut and skin microbiota of yellowtail kingfish. **a** PCoA plot representing Bray-Curtis similarities comparing global bacterial assemblages between sample types (gut, skin and seawater) from fish housed in seacages and those relocated and acclimatised in tanks. **b**-**c** Stacked barplots presenting the mean relative abundance (%) of the top 20 most abundant bacterial ASVs found in the gut of fish and the top 10 bacterial Classes found in the seawater (SW) and on the skin of fish, respectively, comparing fish housed in seacages and those relocated and acclimatised in tanks

In order to evaluate the effects of antibiotics and FMT on the gut and skin bacterial communities of yellowtail kingfish with poor gut health, fish suffering from a putative enteritis were first translocated from an offshore seacage to a series of onshore treatment tanks where they were allowed to acclimatise for 3 weeks prior to commencing the trial. The impact of this change was initially assessed by comparing a subset of fish sampled at the time of collection from the seacage (*n* = 10) to a group of fish sampled from the tanks 3 weeks post acclimatisation (*n* = 12). Ordination of the samples based on Bray-Curtis dissimilarity matrix revealed independent clustering of the skin and gut samples (Fig. 2a) (one-way PERMANOVA: Pseudo-F = 38.3, *p* < 0.01), as expected. However, the acclimatisation process had no effect on the global gut bacterial communities (one-way PERMANOVA: Pseudo-F = 2.5, *p* = 0.063) (Fig. 2a), with both the seacage and tank fish comprising similar mean relative abundances of the most dominant ASVs (namely uncultured *Mycoplasmataceae* and *Allivibrio*) (Fig. 2b). However, for these and many of the other ASVs detected in this study, species level assignment could not be determined. The gut bacterial assemblages of tank fish comprised a number of additional notable ASVs, representing various genera particularly *Brevinema*, *Vibrio,* and an unclassified *Spirochaetaceae*, while seacage fish also comprised *Pseudoalteromonas*, *Cobetia* and *Halomonas* (Fig. 2b). Differential abundance analysis identified a total of 17 ASVs that were differentially abundant, whereby two were enriched in tank fish (namely *Brevinema* and *Allivibrio*) vs. 15 in seacage fish (which included eight that were associated with *Pseudoalteromonas*) (Fig. 2b, Additional file 1: Table S7).

In evaluating the effect of acclimatisation on the skin bacterial communities, a significant difference was linked to acclimatisation (one-way PERMANOVA: Pseudo-F = 14.8, *p* < 0.001, Fig. 2a), as the fish acclimatised in tanks showed a shift in the ratio of *Proteobacteria:Bacteroidetes* (P:B ratio) with the *Gammaproteobacteria* becoming the more dominant Class (Fig. 2c). Specifically, the mean P:B ratio changed from < 0.5 to > 2 after fish were translocated from seacages and acclimatised in tanks for 3 weeks (Additional file 2: Figure S2). The seawater samples taken from the seacage and the tanks comprised similar bacterial assemblages and clustered independently to those taken from the skin and gut (Fig. 2a).

### Effect of antibiotics on the gut and skin microbiota

**Fig. 3:**
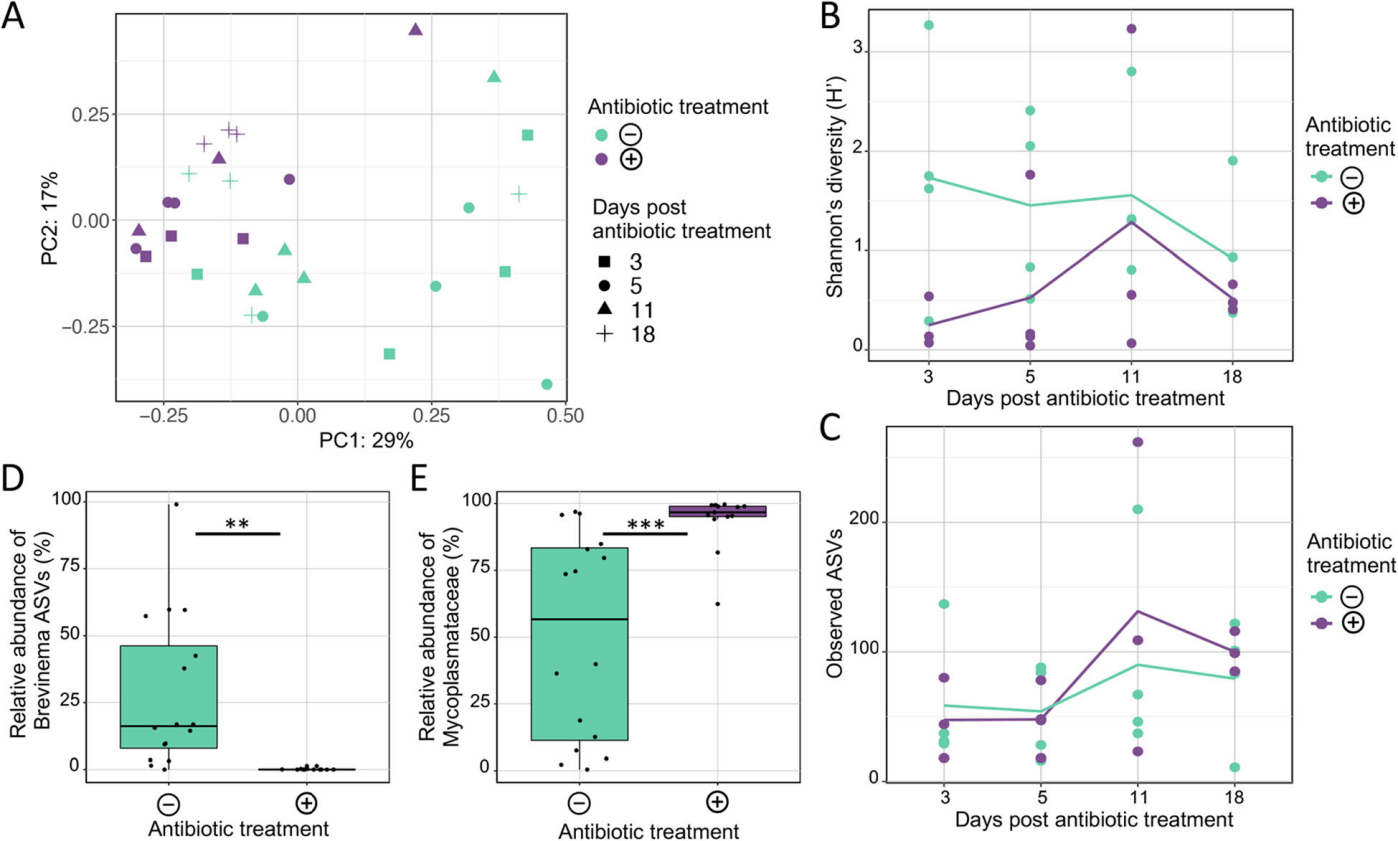
Effect of antibiotics on the gut microbiota of yellowtail kingfish. **a** PCoA plot representing Bray-Curtis similarities comparing the change in global gut bacterial assemblages after treatment with antibiotics (+) over 18-days, with those fish that did not receive treatment (−). **b**-**c** Means plots showing the change in mean value of Shannon’s index of diversity and total observed ASVs (as a measure of richness), respectively, over the 18-day treatment period (from *n* = 4 fish). **d**-**e** Boxplots presenting the median and IQR of the relative abundances of the summed *Brevinema*-associated ASVs and a single ASV associated with *Mycoplasmataceae* (ASV1452), respectively, comparing fish treated and not treated with antibiotics. The level of significant difference is denoted by *p ≤ 0.05, **p ≤ 0.01 or ***p ≤ 0.001, following the Mann-Whitney U test

To explore the impact of the antibiotic combination therapy on the gut and skin associated bacterial communities, fish from two tanks treated with antibiotics were compared with fish from two untreated tanks over a period of 18 days (i.e. at days 3, 5, 11 and 18 days post antibiotic treatment). Antibiotic treatment had a significant effect on the global gut bacterial communities (two-way PERMANOVA: Pseudo-F = 4.4, *p* < 0.001), with no significant difference over time (Pseudo-F = 1.39, *p* = 0.085) and no significant interaction effect between antibiotic treatment and time (Pseudo-F = 0.86, *p* = 0.669) (Fig. 3a), indicating that the antibiotic effect lasted for up to 18 days. This corresponded with a loss of ASV diversity and evenness within the gut bacterial assemblages of fish treated with antibiotics (Fig. 3b and Additional file 2: Figure S3a), as based on measures of Shannon’s diversity (two-way ANOVA: F = 5.36, *p* = 0.029) and Pielou’s evenness (F = 10.98, *p* = 0.003) respectively. No significant differences were observed for these diversity metrics over time, indicating that the community did not recover over the 18-day period (*p* > 0.05). However, this did not correspond to a loss in ASV richness or phylogenetic diversity (two-way ANOVA: *p* > 0.05; Fig. 3c and Additional file 2: Figure S3b), indicating that ASVs were diminished but not completely eliminated following antibiotic treatment. To further explore which microbes were susceptible to the antibiotics, differential abundance analysis of ASVs was performed. Three ASVs were significantly reduced in the gut of antibiotic treated fish: two associated with *Brevinema* (Fig. 3d) and one associated with *Aliivibrio* (Additional file 1: Table S8)*.* Moreover, an unclassified *Mycoplasmataceae* related ASV became substantially more dominant in fish exposed to antibiotic treatment (Fig. 3e). While its abundance in control fish varied considerably, this ASV was always dominant in fish administered antibiotics.

Antibiotic treatment also had a significant impact on the global skin bacterial communities (two-way PERMANOVA: Pseudo-F = 2.74, *p* < 0.01), with the most notable differences occurring up to 3 days post-treatment (Additional file 1: Table S9; Additional file 2: Figure S4). Changes in the global bacterial assemblages were also observed over time, irrespective of antibiotic treatment (two-way PERMANOVA: Pseudo-F = 8.41, *p* < 0.001). Despite some disparity in the clustering of samples from antibiotic treated and control fish, there was a lack of notable difference in the diversity, evenness, richness or phylogenetic diversity, which is likely due to variation observed among fish within some treatment groups (Additional file 2: Figure S5a-S5d). Eight of the total 2672 skin ASVs were significantly more abundant in antibiotic treated fish compared to the control. These included *Tenacibaculum*, *Oleiphilus*, *Glaciecola*, *Paraglaciecola* and an uncultured *Saccharospirillaceae* (Additional file 1: Table S10).

### Impact of FMT on the gut microbiota

**Fig. 4:**
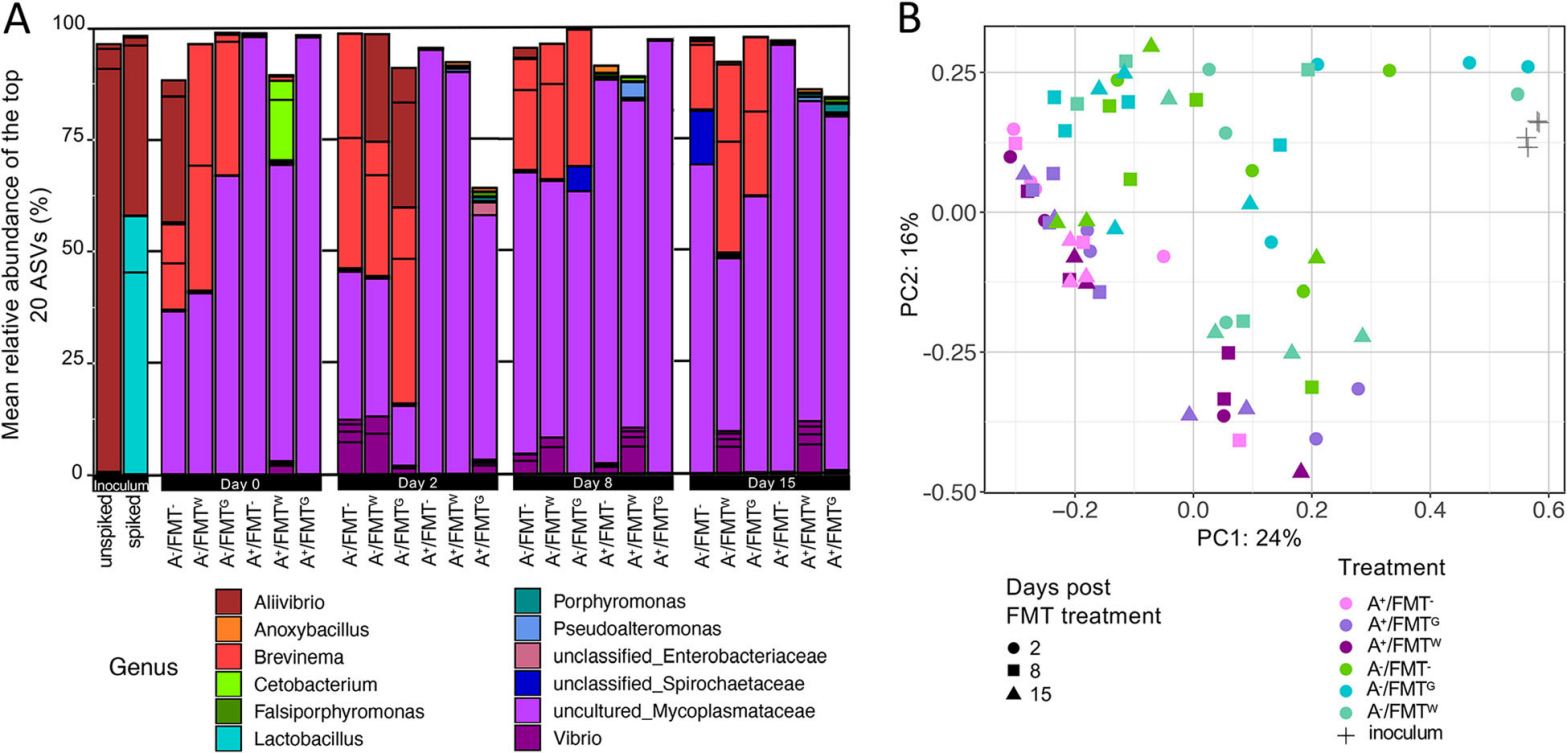
Impact of FMT on antibiotic perturbed and unperturbed gut microbiota. **a** Stacked barplot presenting the mean relative abundance (%) of the top 20 bacterial ASVs found in the healthy donor inoculum compared to the fish from the different treatment groups; A^−^/FMT^−^, A^+^/FMT^−^, A^−^/FMT^G^, A^+^/FMT^G^, A^−^/FMT^W^ and A^+^/FMT^W^. Mean from *n* = 4 fish. **b** PCoA plot representing Bray-Curtis similarities, comparing global gut bacterial communities between the treated (antibiotic and/or FMT) and untreated fish to the donor inoculum, comparing treatment effects for 15 days post FMT

To measure the effect of FMT on antibiotic perturbed and unperturbed gut associated bacterial communities of yellowtail kingfish, fish from 4 tanks administered FMT via gavage and from 4 tanks administered FMT via water bathing were compared with and without antibiotic pre-treatment (2 tanks per treatment group). In addition, these treatment groups were also compared to fish from untreated tanks and tanks that only received the antibiotic treatment (serving as controls). Fish were endpoint sampled at 0, 2, 8 and 15-days post FMT treatment. In addition, half of FMT-treated tanks were spiked with a specific-*Lactobacillus* strain as an internal control (i.e. *L. rhamnosus* GG or LGG®). However, this organism was not established or detected in fish receiving this treatment despite its predominance in the spiked inoculum (Fig. 4a). The unspiked FMT inoculum was also sampled to discern the global catalogue and active bacterial constituents by sampling the DNA and RNA respectively. A total of 95 ASVs were detected from DNA, 41 from RNA, and 27 that were detected in both (Additional file 2: Figure S6). To exclude the influence of the environment (seawater) on FMT treatment, ASVs from the unspiked inoculum were also compared with those from the seawater. Of the 562 ASVs detected in seawater, only 13 occurred in DNA, one in RNA and two occurred in both the DNA and RNA inoculum samples (Additional file 2: Figure S6). These two shared ASVs belonged to an unclassified *Lactobacillus* sp. and *Allivibrio* and were predominant in the unspiked inoculum, with the seawater samples only comprising minute counts, indicating that they likely represent host rather than environmental associated ASVs. The low numbers of host-associated (gut-derived inoculum) ASVs in seawater highlights the independent nature of the sampled niches, and is reflected in the independent clustering of the samples as detailed above and depicted in Fig. 2a.

Fish that received FMT following antibiotic treatment clustered independently away from the donor inoculum as well as to those fish that received FMT without antibiotic treatment, indicating that the antibiotics had a prolonged effect on the global gut bacterial assemblages and FMT establishment (Fig. 4b). This is evident by a clear separation of the A^+^/FMT^G^ and A^+^/FMT^W^ samples (purple shaded symbols) from the A^−^/FMT^G^ and A^−^/FMT^W^ samples (green shaded symbols). There were no significant differences between the global bacterial communities of fish who received FMT via water or gavage to fish who did not receive FMT, in either the antibiotic treated cohort (two-way PERMANOVA: Pseudo-F = 1.23, *p*-value = 0.183) or the non-antibiotic treated cohort (two-way PERMANOVA: Pseudo-F = 0.97, *p*-value> 0.486). However, there was a significant difference in respect to time post FMT in the non-antibiotic treated cohort (two-way PERMANOVA: Pseudo-F = 2.26, *p*-value< 0.01), indicating that any slight modifications to the global bacterial communities in this treatment group were not static. Indeed, the fish gut microbiota was significantly different in T1 compared to both T2 (*p* = 0.018) and T3 (*p* = 0.018) although it did not change from T2 to T3 (*p* = 0.176). In contrast, time had no influence on the bacterial communities on the antibiotic-treated cohort (two-way PERMANOVA: Pseudo-F = 1.34, *p*-value = 0.132).

There was notably high variation between treated fish within the same treatment group/tank. For example, of the four fish sampled from each treatment at Day 2, two fish from the A^−^/FMT^G^ and one fish from the A^−^/FMT^W^ had global gut bacterial assemblages better resembling the donor inoculum as indicated by the three symbols clustering closely to the grey crosses on Fig. 4b. This suggests that FMT treatment via gavage and water did have some impact on some individual fish (despite the PERMANOVA results on the global bacterial communities above indicating that the effect of FMT was minor). To best quantify the effect of FMT on establishment within treated fish, the change in resemblance to the healthy donor inoculum was calculated using the Bray-Curtis (BC) similarity algorithm. This gives a percent similarity between pairs of samples. First, the mean similarity between the 16 control fish that received no antibiotic or FMT treatment to the unspiked inoculum was 10.8% (median of 6.5%). This indicates that the healthy donor fish and the poor performing fish do not share the same ASVs or that the relative ASV abundance of shared ASVs differ vastly between these fish cohorts. At the RNA level, the inoculum was dominated by an ASV associated with *Aliivibrio* (see Fig. 4a), while the remaining ASVs belonged to uncultured *Mycoplasmataceae*, *Brevinema*, *Aliivibrio*, *Vibrio*, and *Lactobacillus*.

**Fig. 5:**
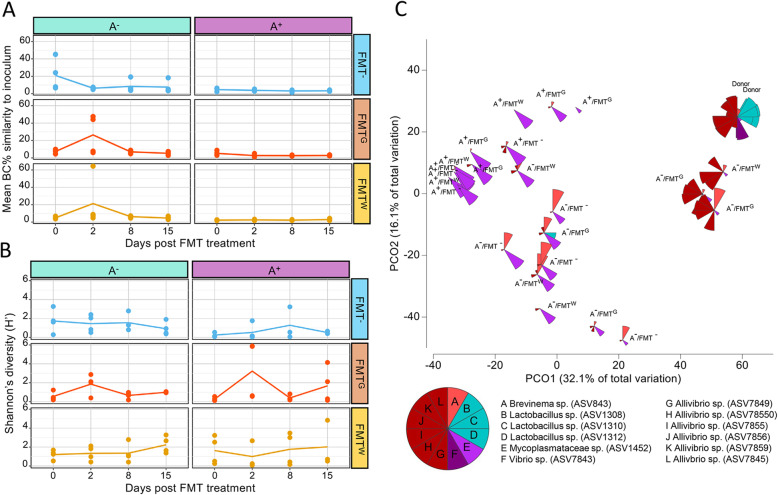
Impact of FMT on antibiotic perturbed and unperturbed gut microbiota. **a**-**b** Line plot presenting the mean Bray-Curtis similarity to the donor inoculum and mean Shannon’s index of diversity, respectively, for the different treatment groups; A^−^/FMT^−^, A^+^/FMT^−^, A^−^/FMT^G^, A^+^/FMT^G^, A^−^/FMT^W^ and A^+^/FMT^W^ over 15 days post FMT (mean from *n* = 4 fish). **c** PCoA plot representing Bray-Curtis similarities, comparing global gut bacterial communities between the treated (antibiotic and/or FMT) and untreated fish to the donor inoculum at Day 2 post FMT, with superimposed wedges indicating the relative abundance of the 12 most abundant ASVs in the RNA component of the donor inoculum. Wedge size is proportional to its rank order across all samples, whereby large wedges indicate those ASVs that had the greatest ranked abundance between samples, and the smallest wedges having the lowest ranked abundance between samples

By comparing the global gut bacterial communities of both treated and untreated fish to the healthy donor inoculum, there was a clear shift in both BC% value and diversity in some treated groups at Day 2 post FMT (Fig. 5a-b). For example, there was an increase in diversity in some fish from the FMT via gavage treatment group, especially in fish treated with antibiotics (A^+^/FMT^G^)(Fig. 5b). This increased diversity was also associated with an increased richness in some individuals (Additional file 2: Figure S7). The two fish with high diversity and richness were sampled from different tanks, excluding a tank effect during the experimental period. As noted above, the fish with greatest similarity to the donor inoculum belonged to the A^−^/FMT^G^ and A^−^/FMT^W^ groups, with similarities to the inoculum of 44–64% (Fig. 5a), a marked difference from the median value of ~ 6% for control fish. However, the other fish within these treatment groups did not have such high similarities to the inoculum (6–8%), suggesting that FMT only works on some animals (Fig. 5a). Furthermore, the lower BC% at later timepoints (8 and 15 days post FMT) indicates that FMT only induced short-lived changes in the bacterial communities.

Of the 79 healthy-donor (RNA/DNA) inoculum ASVs that were not detected in any control fish (A^−^/FMT^−^) or seawater, 17 were detected at least once in the FMT^+^ treatment groups (11 ASVs in the A^−^/FMT^+^ and 6 ASVs in the A^+^/FMT^+^). Sixty-two ASVs were only detected in the inoculum and were not found in any fish from any treatment group or from seawater. A deeper look into those ASVs that were able to be transplanted or enriched at Day 2 post FMT in these fish that resembled the inoculum included several *Aliivibrio* ASVs such as ASV7859, ASV7856, ASV7855, ASV7850 and ASV7849, and a *Lactobacillus* ASV (ASV1312) (Fig. 5c).

## Discussion

In commercial aquaculture operations, marine fish species are raised either in open water seacages or on land in tanks. Inherent variations exist between these systems and may have an impact on the fishes’ associated microbiota. For example, the gut bacterial assemblages of Atlantic salmon (*Salmo salar*) raised in a recirculation system compared to those from open commercial cages, have been shown to vary and are associated with the occurrence of unique species in each system [37]. In this study, however, no significant differences were observed in the global gut bacterial communities between seacage fish and those that were translocated from seacages and allowed to acclimate in tanks for 3 weeks. Given that both groups of fish originated from the same seacage and were maintained on the same pelleted diet, this is not surprising and suggests that any potential stresses imposed on the translocated fish (e.g. transport and variations in water quality) did not impact their gut communities prior to the commencement of the trial. In contrast, the global skin bacterial communities differed between seacage fish and translocated seacage fish acclimated in tanks, where a shift in the ratio of *Proteobacteria:Bacteroidetes* (P:B ratio) was apparent due to the increased abundance of *Gammaproteobacteria* in fish acclimated in tanks. Given the occurrence of similar types of environmental (seawater) bacterial assemblages in both systems, it is likely then that other factors may have contributed to the selection of particular taxa in this instance. This may include factors that contribute to the physiological stress of the animal (e.g. stocking density, current, swimming and oxygen availability), which in turn may impact the way in which they respond and regulate their microbiota [15]. This was recently demonstrated in work reporting on the skin P:B ratio as a biomarker for performance in yellowtail kingfish, where in comparing wild to farmed healthy and diseased seacage fish, low P:B ratios of < 2 were associated with fish with early stages of disease (compared to > 10 in wild) [26]. In this work, the mean P:B ratio changed from < 0.5 to > 2 after fish were translocated from seacages and acclimatised in tanks for 3 weeks and may suggest a positive change in the balance of the bacterial communities. However, further work would be required to elucidate whether this corresponds to improvements in the health status of these fish.

In poor performing yellowtail kingfish in this trial, the gut bacterial assemblages were dominated by a number of ASVs relating to uncultured *Mycoplasmataceae* and *Allivibrio*. Some additional ASVs were more abundant in either tank or seacage fish and included, among others, *Brevinema* in tank fish, and *Pseudoalteromonas* in the seacage fish. As the dominant constituent here in poor performing fish, the occurrence of a single ASV related to an uncultured *Mycoplasmataceae* raises questions around its association with disease. As a member of this bacterial family, *Mycoplasma* have been previously identified in the gut of other fish such as Atlantic salmon, Chinook salmon (*Oncorhynchus tshawytscha)*, zebrafish (*Danio rerio*), common carp (*Cyprinus carpio*), largemouth bronze gudgeon (*Coreius guichenoti*) and rainbow trout [38–44]. While recent genome reconstruction indicates a mutualistic lifestyle of this organism in the intestine of certain species like Atlantic salmon and hadal snailfish (*Pseudoliparis swirei*) [45, 46], for others it has been associated with disease [42]. Members of the genus *Allivibrio* largely form part of the natural gut microbiota of various fish (e.g. cods, Atlantic salmon) [47, 48], though certain species have also been found to be pathogenic (e.g. *A. wodanis*, *A. salmonicida*, *A. fischeri*) [49–51]. *Brevinema* has been found in the gut of Atlantic salmon and Atlantic cod (*Gadus morhua*) [52, 53] as well as in rainbow trout (*Oncorhynchus mykiss*) with genetic susceptibility to particular pathogens (e.g. *Flavobacterium psychrophylum*) [54]. Some members of the genus *Brevinema* found in Atlantic salmon have, however, been reported to produce butyrate [53], which may support intestinal barrier function and mucosal immunity [55, 56]. With other dominant constituents found here (e.g. *Pseudoalteromonas*) also reported to comprise both pathogenic [57] and beneficial (probiotic-like) species [58–61] questions thus remain around their role and changes in abundance between cultivation systems and requires further elucidation, particularly given the inability to resolve many of the ASVs to a species level in this study.

Antibiotic treatment (consisting of a combination therapy comprising oxytetracycline, erythromycin and metronidazole) had a notable impact on these taxa (e.g. uncultured *Mycoplasmataceae* and *Brevinema*), and the bacterial communities more broadly. Specifically, in the gut, a shift in the global bacterial assemblages was evident immediately in response to the treatment and was marked by a loss of species (ASV) diversity and evenness, which did not recover over the 18 day period. Despite this, the species (ASV) richness did not change substantially over this period, indicating that while antibiotic treatment had a significant effect on these assemblages, many of the species were likely diminished but not completely eliminated. This raises questions around whether these populations have the capacity to return to their ‘original’ composition over a prolonged period (in this case beyond 18 days), or whether they are likely to remain in (and continue to evolve from) an altered state after antibiotic treatment. Studies from other fish species have also demonstrated the notable effects that antibiotics may have on the gut microbiota. For example, a loss of gut diversity was observed in Atlantic salmon following oxytetracycline treatment [62], in channel catfish (*Ictalurus punctatus*) following florfenicol treatment [63], in fathead minnow (*Pimephales promelas*) after triclosan use [64] and in zebrafish following olaquindox treatment [65]. In contrast, in some cases, it has been reported that antibiotic treatment may even increase or cause a shift in species diversity, as shown for Atlantic salmon and zebrafish [12, 13], and pacu (*Piaractus mesopotamicus*) [8] respectively. This is pertinent given that changes in diversity and evenness within the gut has been suggested to influence functional capacity and disease resilience [66]. While contentious, the consequences of this may be significant, particularly where the communities fail to recover over an extended period, as observed here. Attempts to investigate the functional changes in these communities in response to antibiotic treatment (e.g. using metagenomics or metatranscriptomics) is thus warranted.

Antibiotic treatment also had a significant impact on the global skin bacterial communities, with the most notable differences occurring up to 3 days post-treatment. Unlike the gut though, global changes were also observed to occur with time (irrespective of antibiotic treatment), indicating that while antibiotic treatment may have immediate, broader effects outside of the gut, the skin communities are also inherently more dynamic. This was further exemplified by the notable variation observed between individuals, which obscured any apparent differences in the diversity, evenness, richness or phylogenetic diversity. Instead, several ASVs were found to be significantly enriched in the skin of antibiotic treated fish, particularly *Tenacibaculum.* This is a concern, as this genus encompasses numerous pathogenic species which have the capacity to cause serious ulcerative disease (tenacibaculosis) in a wide range of marine fish species [67–69]. In other fish, treatment with antibiotics has also been shown to have negative effects that extend across the mucosal surfaces. For example, rifampicin exposure (via bathing rather than oral administration) led to the reduction of both the skin and gut associated microbial diversity in western mosquitofish (*Gambusia affinis*) [70] and led to an increase in the susceptibility to certain opportunistic pathogens and stressors, and a failure to thrive over a prolonged period. Furthermore, it was shown that while these communities stabilise during recovery, they do not appear to return to their original state in the short-term (~ 1 week). In some fish such as sea bass (*Dicentrarchus labrax*), it has also been shown that recovery over the longer term (~ 3 weeks following oral administration of oxytetracycline) may vary between the different mucosal surfaces, with the communities associated with the skin reported to be more resilient to those of the gills [71]. In support of this, we also observed here a greater disparity in the effects of antibiotic treatment on the gut rather than the skin bacterial communities. While we cannot exclude the possibility that variations in dosing may have contributed to this finding (e.g. from partial or complete regurgitation of the administered antibiotics), it is likely that this was due to the mode in which the antibiotics were delivered, whereby in this case initial exposure and uptake occurred first in the gut followed by its subsequent dissemination through the body and into the outer mucosal surfaces. Further variations in the specific pharmacokinetics of the antibiotics, however, may also be a contributing feature, particularly given the low level of absorption (< 3% of the administrated dose) reported for antibiotics like oxytetracycline in other fish species [72]. The approach to antibiotic administration and treatment should thus be extended to include varied and alternative dosing regimens.

To further assess for which taxa were affected by the antibiotics, differential abundance analysis was performed, revealing three ASVs that were significantly reduced in the gut of antibiotic treated fish (two associated with *Brevinema* and one associated with *Aliivibrio*) and one that became substantially more dominant (namely an unclassified *Mycoplasmataceae* sp.). As stated above, the antibiotic treatment administered in this study comprised a combination of agents (namely oxytetracycline, erythromycin and metronidazole), which together have the capacity to target a wide range of both gram-positive and gram-negative organisms. Given that both *Brevinema* and *Allivibrio* are gram-negative (microaerophilic or facultative anaerobic) bacteria [73, 74], their depletion following treatment was not surprising. What was unexpected, however, was the increase in dominance of an ASV representing an unclassified *Mycoplasmataceae* species. As a member of this bacterial family, *Mycoplasma* are characterised by a lack of cell wall around their membrane which makes them resistant to antibiotics targeting cell wall synthesis such as beta-lactams, glycopeptides and fosfomycin [75]. However, oxytetracycline (a tetracycline) is known to be an effective treatment for *Mycoplasma* infections as it targets protein rather than cell wall synthesis [76]. In addition, erythromycin (a macrolide) and metronidazole (a nitroimidazole) are also both inhibiters of protein synthesis [77, 78], thus their mode of action should presumably have contributed to the depletion (rather than the increase in abundance) of *Mycoplasma*. Despite this, it has been found that this genus can quickly develop resistance to both macrolides and tetracyclines [79]. While it is tempting to postulate then that such mechanisms may have led to its increase in abundance here, it is important to note that it could have equally been depleted following antibiotics but remained at a high relative abundance because of the depletion of other taxa. Nevertheless, given the inherent parasitic nature of the *Mycoplasma* [80], it would be prudent to further investigate changes in their actual abundance in response to antibiotic treatment (e.g. using qPCR) in a farm setting more broadly, as well as the likely resistance mechanisms encoded within its genome, and would support an improved understanding of its role in yellowtail kingfish health.

Since antibiotic exposure can perturb the microbiota and may have possible consequences for the health of the animal, attempts have been made in helping the microbiota recover to re-establish homeostasis. Traditionally, this has included, among others, the use of various probiotic microorgansims. For instance, within black molly (*Poecilia sphenops*) the administration of native probiotics (namely *Phaeobacter inhibens* and *Bacillus pumilus*) following antibiotic exposure led to improved disease resistance to pathogenic *Vibrio* species [81]. In this study, we attempted to introduce a purported probiotic *Lactobacillus* species (*L. rhamnosus* GG or LGG®, of human origin) in conjunction with FMT, which was previously used to improve disease resistance and the immune response in other fish species [82]. However, this organism was not detected in any of the samples here. While this suggests that this strain may thus not be able to colonise the mucosal surfaces of yellowtail kingfish, further validation using more sensitive approaches like qPCR would be required. Despite this, other differentiable *Lactobacillus* related ASVs were detected in the gut samples following FMT treatment, indicating that these organisms may naturally occur in yellowtail kingfish as part of a broader group of other lactic acid bacteria (LAB) reported in finfish [83]. In this regard, the use of autochthonous probiotics for this species would be more appropriate and would likely improve the prospect of successful establishment within the gastrointestinal tract. Recently, a total of 11 isolates (including members of *Shewanella*, *Psychrobacter*, *Acinetobacter*) from yellowtail kingfish was discovered but further work is required to evaluate their potential benefits in the farming of this species [84].

As an alternative, more holistic biological approach for modulating the gut microbiota, FMT was also investigated in this study and was administered to a total of 96 poor-performing yellowtail kingfish. Alongside groups of fish that solely received or were administered FMT following antibiotic pre-treatment, two approaches to FMT were evaluated. As strategies used previously for fish [24, 85, 86], this included the direct delivery of a single FMT inoculum via oral gavage, and the indirect delivery of this inoculum via bathing in a reduced volume of seawater for a prolonged period (~3 hours). To elucidate the effects of FMT and the approach to its administration, end-point samples of both the gut and skin were evaluated over a 15-day period (i.e. at 0, 2, 8 and 15-days post FMT administration). Although no significant differences in the global bacterial communities were observed between the FMT treated and control groups of fish (regardless of the route of administration), any broad effect of FMT was masked by the notable variation apparent between individuals. Despite this, samples from several of the oral-gavage and seawater bathed FMT-treated fish appeared to cluster more closely to the FMT donor inoculum samples, indicating some level of impact. Indeed, for some fish, a similarity with the donor inoculum of up to 64% was observed (compared to only ~ 6% for the control fish) and was most notable at Day 2 post FMT treatment. Much lower similarities were observed, however, at the later time points (i.e. at 8 and 15 days post FMT delivery) and for many of the other fish, thus indicating that FMT may only induce short-lived changes in certain individuals. While it is unclear why this was observed, likely explanations may include variations in the “colonisation resistance” of the respective gut communities to the introduction of exogenous microorganisms; a feature that has been suggested for humans and rodents and purported to be exacerbated by treatment with antibiotics [87, 88]. Further investigations into the mechanisms that contribute to resilience would thus be pertinent for improving the efficacy of FMT.

While it is not possible to completely exclude variations in the initial composition of the microbiota between fish in the individual treatment groups (due to end-point sampling), the gut bacterial assemblages of the poor-performing control fish at the beginning of the trial were markedly different to those from the healthy donor inoculum (as derived from 102 healthy seacage fish), and suggests that other factors may have contributed to the higher similarities observed for these select individuals. As postulated in studies using FMT to modulate the gut microbiota of killifish [24], such findings may also include variations in the fish’s immune response and its capacity to influence establishment. Furthermore, genetic diversity is also known to shape the selection of the host microbiota [89–91], potentially resulting in the varied responses to FMT among the same population of fish. Considering that these fish came from a cohort comprising mixed genetics, this may in part also explain why only a small proportion of the total ASVs detected in the inoculum (i.e. 17/79) were observed in the FMT treated fish and may reflect a limitation of the current approach. Nevertheless, given that certain dominant constituents prone to the antibiotic treatment were able to be transferred to select individuals (e.g. *Allivibrio* spp.), this suggests that FMT has some capacity to influence the microbiota (irrespective of whether it is delivered directly or indirectly) and warrants further investigation. This should include strategies used elsewhere to improve and prolong its effects in other animals, e.g. through the administration of multiple consecutive doses of the FMT inoculum and by pre-treating the inoculum to support the survival of potentially fastidious constituents [92] or by using material derived from wild (rather than healthy farmed) individuals for restoring potentially ‘extinct’ autochthonous taxa [93]. In addition, given the profound impacts FMT mediated gut microbiota alterations can have on the animal’s health (as recently demonstrated in Pacific white shrimp, *Litopenaeus vannamei* [94]), further work is also required to elucidate the role of FMT in modulating the health outcomes in yellowtail kingfish.

## Conclusion

In this study, the impacts of antibiotics on the gut and skin microbiota of a commercially important farmed finfish species *Seriola lalandi* (yellowtail kingfish) was investigated. The oral administration of a broad-spectrum antibiotic combination therapy in poor-performing fish significantly perturbed the global gut and skin bacterial assemblages and led to the loss of key constituents and the concomitant enrichment of potentially opportunistic species. Unlike the gut where a prolonged effect was observed, the bacterial assemblages of the skin appeared to be dynamic and inherently more resilient to the treatment (likely due to the varied pharmokinetics of the compounds), and shifted towards a more ‘normal’ (though disparate) state over the recovery period. Given the increasing awareness of the role the microbiota plays in supporting host health, attempts to restore changes exerted by the antibiotic treatment on these bacterial communities was undertaken using faecal microbiota transplantation (FMT). As derived from a population of healthy farmed yellowtail kingfish, FMT was delivered both directly (via oral gavage) and indirectly (through the surrounding seawater). Despite the lack of notable global changes in the gut bacterial communities in FMT treated fish, for some individuals the effect was profound (regardless of the mode of delivery) and was associated with a change in the bacterial composition that more closely resembled that of the donor inoculum. Though short-lived, this suggests the potential of FMT for modulating these communities. Further work is required, however, to improve the approach (e.g. using more varied, pre-screened inoculums) and to evaluate its capacity to influence health in yellowtail kingfish. To this end, metagenomic and/or metatranscriptomic methods would represent useful tools for supporting an improved understanding of the global functional changes in these communities in response to these treatment regimens.

## Supplementary information

**Additional file 1: Table S1.** Water temperature (°C) recorded during the experiment. **Table S2:** Oxygen concentration (%) recorded during the experiment. **Table S3:** pH recorded during the experiment. **Table S4:** Salinity (‰) recorded during the experiment. **Table S5:** Ammonia concentration (ppm) recorded during the experiment. **Table S6:** Characteristics (weight and length) of the fish stocked in the 12 experimental tanks prior the start of the experiment. **Table S7:** Differential abundant ASVs found in the gut microbiota of fish housed in seacage and tanks. **Table S8:** Differential abundant ASVs found in the gut microbiota of fish treated with antibiotic and non-treated with antibiotic. **Table S9:** Pair-wise PERMANOVA results investigating the influence of time on the skin microbial communities. **Table S10:** Differential abundant ASVs found in the skin microbiota of fish treated with antibiotic and non-treated with antibiotic.

**Additional file 2: Figure S1.** Rarefaction plot of all samples analysed in this study. **Figure S2:** Stacked barplots presenting the mean relative abundance (%) of the top 10 bacterial Phylum found in the seawater (SW) and on the skin of fish, comparing fish housed in seacages and those relocated and acclimatised in tanks. **Figure S3:** Means plots showing the change in mean value of Pielou’s evenness (a) and Faith’s phylogenetic diversity (b) in the gut bacterial communities, over the 18-day treatment period (from *n* = 4 fish). **Figure S4:** PCoA plot representing Bray-Curtis similarities comparing the change in global skin bacterial assemblages after treatment with antibiotics (+) over 18-days, with those fish that did not receive treatment (−). **Figure S5:** Means plots showing the change in mean value of Shannon’s index of diversity (a), Pielou’s evenness (b), total observed ASVs (as a measure of richness) (c) and Faith’s phylogenetic diversity (d) in the skin bacterial communities, over the 18-day treatment period (from *n* = 4 fish). **Figure S6:** Venn diagram showing the distribution of unique and shared ASVs in the seawater, and the DNA and RNA inoculum samples. The total number of ASVs within each group are denoted in parentheses. **Figure S7:** Mean plot presenting the mean number of observed ASVs for the different treatment groups; A^−^/FMT^−^, A^+^/FMT^−^, A^−^/FMT^G^, A^+^/FMT^G^, A^−^/FMT^W^ and A^+^/FMT^W^ over 15 days post FMT (mean from *n* = 4 fish).

## Data Availability

Sequencing reads from the demultiplexed samples analysed in this study have been deposited in the NCBI Sequence Read Archive (SRA) under the BioProject accession PRJNA602789. Scripts used to analyse the 16S rRNA gene sequence data using phyloseq have been deposited in GitHub at https://github.com/aoxley1975/Kingfish

## Abbreviations

16S rRNA: 16 Svedberg ribosomal ribonucleic acid
ARG: Antimicrobial resistant gene
ASV: Amplicon sequence variant
cDNA: Complementary DNA
FMT: Faecal microbiota transplantation
NCBI: National Centre for Biotechnology Information
PCoA: Principle Coordinate Analysis
PCR: Polymerase Chain Reaction
PERMANOVA: Permutational Multivariate Analysis Of Variance

## References

[1] FAO (2018). The State of World Fisheries and Aquaculture 2018-Meeting the sustainable development goals.

[2] Defoirdt T, Sorgeloos P, Bossier P (2011). Alternatives to antibiotics for the control of bacterial disease in aquaculture. Curr Opin Microbiol.

[3] Pérez-Sánchez T, Mora-Sánchez B, Balcázar JL. Biological approaches for disease control in aquaculture: advantages, limitations and challenges. Trends Microbiol. 2018;26(11):896–903. 10.1016/j.tim.2018.05.002 29801773

[4] Lulijwa R, Rupia EJ, Alfaro AC (2019). Antibiotic use in aquaculture, policies and regulation, health and environmental risks: a review of the top 15 major producers. Rev Aquac.

[5] Miranda CD, Godoy FA, Lee MR (2018). Current status of the use of antibiotics and the antimicrobial resistance in the Chilean Salmon farms. Front Microbiol.

[6] Henriksson PJG, Rico A, Troell M, Klinger DH, Buschmann AH, Saksida S, Chadag MV, Zhang WB (2018). Unpacking factors influencing antimicrobial use in global aquaculture and their implication for management: a review from a systems perspective. Sustain Sci.

[7] Higuera-Llantén S, Vasquez-Ponce F, Barrientos-Espinoza B, Mardones F, Marshall S, Olivares-Pacheco J. Extended antibiotic treatment in salmon farms select multiresistant gut bacteria with a high prevalence of antibiotic resistance genes. PLoS One. 2018;13(9):22. 10.1371/journal.pone.0203641 PMC6133359 30204782

[8] Sáenz JS, Marques TV, Barone RSC, Cyrino JEP, Kublik S, Nesme J, Schloter M, Rath S, Vestergaard G. Oral administration of antibiotics increased the potential mobility of bacterial resistance genes in the gut of the fish *Piaractus mesopotamicus*. Microbiome. 2019;7:14. 10.1186/s40168-019-0632-7 PMC6378726 30773139

[9] Shen YB, Zhou HW, Xu J, Wang YQ, Zhang QJ, Walsh TR, Shao B, Wu CM, Hu YY, Yang L, et al. Anthropogenic and environmental factors associated with high incidence of *mcr*-1 carriage in humans across China. Nat Microbiol. 2018;3(9):1054–62. 10.1038/s41564-018-0205-8 PMC6198934 30038311

[10] Zeng Q, Liao C, Terhune J, Wang L (2019). Impacts of florfenicol on the microbiota landscape and resistome as revealed by metagenomic analysis. Microbiome.

[11] Vincent AT, Gauthier J, Derome N, Charette SJ (2019). The rise and fall of antibiotics in aquaculture. Microbial communities in aquaculture ecosystems.

[12] Gupta S, Fernandes J, Kiron V (2019). Antibiotic-induced perturbations are manifested in the dominant intestinal bacterial phyla of Atlantic Salmon. Microorganisms.

[13] López Nadal A, Peggs D, Wiegertjes GF, Brugman S (2018). Exposure to antibiotics affects Saponin immersion-induced immune stimulation and shift in microbial composition in Zebrafish larvae. Front Microbiol.

[14] Ferrer M, Méndez-García C, Rojo D, Barbas C, Moya A. Antibiotic use and microbiome function. Biochem Pharmacol. 2017;134:114–26. 10.1016/j.bcp.2016.09.007 27641814

[15] Reese AT, Cho EH, Klitzman B, Nichols SP, Wisniewski NA, Villa MM, Durand HK, Jiang S, Midani FS, Nimmagadda SN (2018). Antibiotic-induced changes in the microbiota disrupt redox dynamics in the gut. eLife.

[16] Legrand TPRA, Wynne JW, Weyrich LS, Oxley APA (2019). A microbial sea of possibilities: current knowledge and prospects for an improved understanding of the fish microbiome. Rev Aquac.

[17] Carnevali O, Maradonna F, Gioacchini G (2017). Integrated control of fish metabolism, wellbeing and reproduction: the role of probiotic. Aquaculture.

[18] Brugman S, Ikeda-Ohtsubo W, Braber S, Folkerts G, Pieterse CM, Bakker PA (2018). A comparative review on microbiota manipulation: lessons from fish, plants, livestock, and human research. Front Nutr.

[19] Jin Song S, Woodhams DC, Martino C, Allaband C, Mu A, Javorschi-Miller-Montgomery S, Suchodolski JS, Knight R (2019). Engineering the microbiome for animal health and conservation. Exp Biol Med.

[20] Eiseman B, Silen W, Bascom GS, Kauvar AJ. Fecal enema as an adjunct in the treatment of pseudomembranous enterocolitis. Surgery. 1958;44(5):854–9. 13592638

[21] Sbahi H, Di Palma JA (2016). Faecal microbiota transplantation: applications and limitations in treating gastrointestinal disorders. BMJ Open Gastroenterol.

[22] Suez J, Zmora N, Zilberman-Schapira G, Mor U, Dori-Bachash M, Bashiardes S, Zur M, Regev-Lehavi D, Brik RB, Federici S (2018). Post-antibiotic gut mucosal microbiome reconstitution is impaired by probiotics and improved by autologous FMT. Cell.

[23] Metzler-Zebeli BU, Siegerstetter SC, Magowan E, Lawlor PG, O'Connell NE, Zebeli Q. Fecal microbiota transplant from highly feed efficient donors affects cecal physiology and microbiota in low- and high-feed efficient chickens. Front Microbiol. 2019;10:13. 10.3389/fmicb.2019.01576 PMC6629952 31354670

[24] Smith P, Willemsen D, Popkes M, Metge F, Gandiwa E, Reichard M, Valenzano DR (2017). Regulation of life span by the gut microbiota in the short-lived African turquoise killifish. eLife.

[25] Sheppard ME. Aquatic animal health subprogram: detection and Management of Health Issues in yellowtail kingfish (YTK, *Seriola lalandi*); the Foundation for a Health Program for Australian finfish aquaculture**;** Final Report: Fisheries Research and Development Corporation; 2005.

[26] Bansemer MS, Forder REA, Howarth GS, Suitor GM, Bowyer J, Stone DAJ (2015). The effect of dietary soybean meal and soy protein concentrate on the intestinal mucus layer and development of subacute enteritis in yellowtail kingfish (Seriola lalandi) at suboptimal water temperature. Aquac Nutr.

[27] Legrand TPRA, Catalano SR, Wos-Oxley ML, Stephens F, Landos M, Bansemer MS, Stone DAJ, Qin JG, Oxley APA (2018). The inner workings of the outer surface: skin and gill microbiota as indicators of changing gut health in yellowtail kingfish. Front Microbiol.

[28] He SX, Ran C, Qin CB, Li SN, Zhang HL, de Vos WM, Ringø E, Zhou ZG. Anti-infective effect of adhesive probiotic *Lactobacillus* in fish is correlated with their spatial distribution in the intestinal tissue. Sci Rep. 2017;7:12. 10.1038/s41598-017-13466-1 PMC5643340 29038557

[29] Zhang JJ, Kobert K, Flouri T, Stamatakis A (2014). PEAR: a fast and accurate Illumina paired-end reAd mergeR. Bioinformatics.

[30] Bolyen E, Rideout JR, Dillon MR, Bokulich N, Abnet CC, Al-Ghalith GA, Alexander H, Alm EJ, Arumugam M, Asnicar F (2019). Reproducible, interactive, scalable and extensible microbiome data science using QIIME 2. Nat Biotechnol.

[31] Callahan BJ, McMurdie PJ, Rosen MJ, Han AW, Johnson AJA, Holmes SP (2016). DADA2: high-resolution sample inference from Illumina amplicon data. Nat Methods.

[32] Quast C, Pruesse E, Yilmaz P, Gerken J, Schweer T, Yarza P, Peplies J, Glöckner FO. The SILVA ribosomal RNA gene database project: improved data processing and web-based tools. Nucleic Acids Res. 2013;41(D1):D590–6. 10.1093/nar/gks1219 PMC3531112 23193283

[33] McMurdie PJ, Holmes S (2013). phyloseq: an R package for reproducible interactive analysis and graphics of microbiome census data. PLoS One.

[34] Anderson MJ (2001). A new method for non-parametric multivariate analysis of variance. Austral Ecol.

[35] Love MI, Huber W, Anders S (2014). Moderated estimation of fold change and dispersion for RNA-seq data with DESeq2. Genome Biol.

[36] Weiss S, Xu ZZ, Peddada S, Amir A, Bittinger K, Gonzalez A, Lozupone C, Zaneveld JR, Vázquez-Baeza Y, Birmingham A, et al. Normalization and microbial differential abundance strategies depend upon data characteristics. Microbiome. 2017;5:18. 10.1186/s40168-017-0237-y PMC5335496 28253908

[37] Dehler CE, Secombes CJ, Martin SAM. Environmental and physiological factors shape the gut microbiota of Atlantic salmon parr (*Salmo salar* L.). Aquaculture. 2017;467:149–57. 10.1016/j.aquaculture.2016.07.017 PMC5142738 28111483

[38] Li XM, Yan QY, Ringø E, Wu XB, He YF, Yang DG. The influence of weight and gender on intestinal bacterial community of wild largemouth bronze gudgeon (*Coreius guichenoti*, 1874). BMC Microbiol. 2016;16:8. 10.1186/s12866-016-0809-1 PMC4994167 27549138

[39] Llewellyn MS, McGinnity P, Dionne M, Letourneau J, Thonier F, Carvalho GR, Creer S, Derome N (2016). The biogeography of the Atlantic salmon (Salmo salar) gut microbiome. ISME J.

[40] Lowrey L, Woodhams DC, Tacchi L, Salinas I. Topographical mapping of the rainbow trout (*Oncorhynchus mykiss*) microbiome reveals a diverse bacterial community with antifungal properties in the skin. Appl Environ Microbiol. 2015;81(19):6915–25. 10.1128/AEM.01826-15 PMC4561705 26209676

[41] Abdelrahman H, ElHady M, Alcivar-Warren A, Allen S, Al-Tobasei R, Bao LS, Beck B, Blackburn H, Bosworth B, Buchanan J (2017). Aquaculture genomics, genetics and breeding in the United States: current status, challenges, and priorities for future research. BMC Genomics.

[42] Gaulke CA, Martins ML, Watral VG, Humphreys IR, Spagnoli ST, Kent ML, Sharpton TJ. A longitudinal assessment of host-microbe-parasite interactions resolves the zebrafish gut microbiome's link to *Pseudocapillaria tomentosa* infection and pathology. Microbiome. 2019;7:16. 10.1186/s40168-019-0622-9 PMC6346533 30678738

[43] Fu PP, Xiong F, Feng WW, Zou H, Wu SG, Li M, Wang GT, Li WX (2019). Effect of intestinal tapeworms on the gut microbiota of the common carp, *Cyprinus carpio*. Parasit Vectors.

[44] Ciric M, Waite D, Draper J, Jones JB. Characterization of mid-intestinal microbiota of farmed Chinook salmon using 16S rRNA gene metabarcoding. Arch Biol Sci. 2019;(00):40.

[45] Jin Y, Angell IL, Rød Sandve S, Snipen LG, Olsen Y, Rudi K. Atlantic salmon raised with diets low in long-chain polyunsaturated n-3 fatty acids in freshwater have a mycoplasma-dominated gut microbiota at sea. Aquac Environ Interact. 2019;11:31–9.

[46] Lian C-A, Yan G-Y, Huang J-M, Danchin A, Wang Y, He L-S. Genomic Characterization of a novel gut symbiont from the Hadal snailfish. Front Microbiol. 2020;10(2978). 10.3389/fmicb.2019.02978 PMC6965317 31998265

[47] Riiser ES, Haverkamp TH, Varadharajan S, Borgan Ø, Jakobsen KS, Jentoft S, Star B. Metagenomic shotgun analyses reveal complex patterns of intra-and interspecific variation in the intestinal microbiomes of codfishes. Appl Environ Microbiol. 2020. 10.1128/AEM.02788-19 PMC7054092 31953333

[48] Wang C, Sun GX, Li SS, Li X, Liu Y. Intestinal microbiota of healthy and unhealthy Atlantic salmon *Salmo salar* L. in a recirculating aquaculture system. J Oceanol Limnol. 2018;36(2):414–26.

[49] Hjerde E, Karlsen C, Sorum H, Parkhill J, Willassen NP, Thomson NR. Co-cultivation and transcriptome sequencing of two co-existing fish pathogens *Moritella viscosa* and *Aliivibrio wodanis*. BMC Genomics. 2015;16:13. 10.1186/s12864-015-1669-z PMC4462113 26059548

[50] Hjerde E, Lorentzen MS, Holden MT, Seeger K, Paulsen S, Bason N, Churcher C, Harris D, Norbertczak H, Quail MA. The genome sequence of the fish pathogen *Aliivibrio salmonicida* strain LFI1238 shows extensive evidence of gene decay. BMC Genomics. 2008;9(1):616. 10.1186/1471-2164-9-616 PMC2627896 19099551

[51] López JR, Lorenzo L, Alcantara R, Navas J. Characterization of *Aliivibrio fischeri *strains associated with disease outbreak in brill *Scophthalmus rhombus*. Dis Aquat Org. 2017;124(3):215–22. 10.3354/dao03123 28492177

[52] Riiser ES, Haverkamp THA, Borgan Ø, Jakobsen KS, Jentoft S, Star B. A single vibrionales 16S rRNA oligotype dominates the intestinal microbiome in two geographically separated Atlantic cod populations. Front Microbiol. 2018;9:14. 10.3389/fmicb.2018.01561 PMC6053498 30057577

[53] Gupta S, Lokesh J, Abdelhafiz Y, Siriyappagouder P, Pierre R, Sørensen M, Fernandes JMO, Kiron V. Macroalga-derived alginate oligosaccharide alters intestinal bacteria of Atlantic Salmon. Front Microbiol. 2019;10:15. 10.3389/fmicb.2019.02037 PMC6753961 31572312

[54] Brown RM, Wiens GD, Salinas I. Analysis of the gut and gill microbiome of resistant and susceptible lines of rainbow trout (*Oncorhynchus mykiss*). Fish Shellfish Immunol. 2019;86:497–506. 10.1016/j.fsi.2018.11.079 PMC8040288 30513381

[55] Canani RB, Di Costanzo M, Leone L, Pedata M, Meli R, Calignano A (2011). Potential beneficial effects of butyrate in intestinal and extrainitestinal diseases. World J Gastroenterol.

[56] Liu H, Wang J, He T, Becker S, Zhang GL, Li DF, Ma X (2018). Butyrate: a double-edged sword for health?. Adv Nutr.

[57] Pujalte MJ, Sitjà-Bobadilla A, Macián MC, Álvarez-Pellitero P, Garay E. Occurrence and virulence of *Pseudoalteromona*s spp. in cultured gilthead sea bream (*Sparus aurata* L.) and European sea bass (*Dicentrarchus labrax* L.). molecular and phenotypic characterisation of P-undina strain U58. Aquaculture. 2007;271(1–4):47–53.

[58] Holmström C, Kjelleberg S. Marine *Pseudoalteromonas* species are associated with higher organisms and produce biologically active extracellular agents. FEMS Microbiol Ecol. 1999;30(4):285–93. 10.1111/j.1574-6941.1999.tb00656.x 10568837

[59] Offret C, Desriac F, Le Chevalier P, Mounier J, Jégou C, Fleury Y. Spotlight on antimicrobial metabolites from the marine bacteria *Pseudoalteromonas*: chemodiversity and ecological significance. Mar Drugs. 2016;14(7):26. 10.3390/md14070129 PMC4962019 27399731

[60] Richards GP, Watson MA, Needleman DS, Uknalis J, Boyd EF, Fay JP. Mechanisms for *Pseudoalteromonas piscicida*-induced killing of Vibrios and other bacterial pathogens. Appl Environ Microbiol. 2017;83(11):17. 10.1128/AEM.00175-17 PMC5440704 28363962

[61] Wesseling W, Lohmeyer M, Wittka S, Bartels J, Kroll S, Soltmann C, Kegler P, Kunzmann A, Neumann S, Ramsch B. Adverse effects of immobilised *Pseudoalteromonas* on the fish pathogenic *Vibrio anguillarum*: an in vitro study. J Mar Biol. 2016;2016.

[62] Navarrete P, Mardones P, Opazo R, Espejo R, Romero J (2008). Oxytetracycline treatment reduces bacterial diversity of intestinal microbiota of Atlantic salmon. J Aquat Anim Health.

[63] Wang EL, Yuan ZH, Wang KY, Gao DY, Liu ZJ, Liles MR (2019). Consumption of florfenicol-medicated feed alters the composition of the channel catfish intestinal microbiota including enriching the relative abundance of opportunistic pathogens. Aquaculture.

[64] Narrowe AB, Albuthi-Lantz M, Smith EP, Bower KJ, Roane TM, Vajda AM, Miller CS (2015). Perturbation and restoration of the fathead minnow gut microbiome after low-level triclosan exposure. Microbiome.

[65] He SX, Wang QM, Li SN, Ran C, Guo XZ, Zhang Z, Zhou ZG (2017). Antibiotic growth promoter olaquindox increases pathogen susceptibility in fish by inducing gut microbiota dysbiosis. Sci China-Life Sci.

[66] De Schryver P, Vadstein O (2014). Ecological theory as a foundation to control pathogenic invasion in aquaculture. ISME J.

[67] Småge SB, Frisch K, Vold V, Duesund H, Brevik ØJ, Olsen RH, Sjaatil ST, Klevan A, Brudeseth B, Watanabe K. Induction of tenacibaculosis in Atlantic salmon smolts using *Tenacibaculum finnmarkense* and the evaluation of a whole cell inactivated vaccine. Aquaculture. 2018;495:858–64.

[68] Bridel S, Olsen A-B, Nilsen H, Bernardet J-F, Achaz G, Avendaño-Herrera R, Duchaud E. Comparative genomics of *Tenacibaculum dicentrarch**i* and “*Tenacibaculum finnmarkense*” highlights intricate evolution of fish-pathogenic species. Genome Biol Evol. 2018;10(2):452–7. 10.1093/gbe/evy020 PMC5793721 29360975

[69] Pérez-Pascual D, Lunazzi A, Magdelenat G, Rouy Z, Roulet A, Lopez-Roques C, Larocque R, Barbeyron T, Gobet A, Michel G. The complete genome sequence of the fish pathogen *Tenacibaculum maritimum* provides insights into virulence mechanisms. Front Microbiol. 2017;8:1542. 10.3389/fmicb.2017.01542 PMC5561996 28861057

[70] Carlson JM, Hyde ER, Petrosino JF, Manage ABW, Primm TP. The host effects of *Gambusia affinis* with an antibiotic-disrupted microbiome. Comp Biochem Physiol C-Toxicol Pharmacol. 2015;178:163–8. 10.1016/j.cbpc.2015.10.004 26475244

[71] Rosado D, Xavier R, Severino R, Tavares F, Cable J, Pérez-Losada M. Effects of disease, antibiotic treatment and recovery trajectory on the microbiome of farmed sea bass (*Dicentrarchus labrax*). Sci Rep. 2019;9:11. 10.1038/s41598-019-55314-4 PMC6908611 31831775

[72] Rogstad A, Hormazabal V, Rasmussen KE, Ellingsen OF (1991). Pharmacokinetic study of oxytetracycline in fish. I. Absorption, distribution and accumulation in rainbow trout in freshwater. Aquaculture.

[73] Defosse D, Johnson R, Paster B, Dewhirst F, Fraser G. *Brevinema andersonii* gen. nov., sp. nov., an infectious spirochete isolated from the short-tailed shrew (*Blarina brevicauda*) and the white-footed mouse (*Peromyscus leucopu*s). Int J Syst Evol Microbiol. 1995;45(1):78–84. 10.1099/00207713-45-1-78 7857811

[74] Beaz-Hidalgo R, Doce A, Balboa S, Barja JL, Romalde JL. *Aliivibrio finisterrensis *sp. nov., isolated from Manila clam, Ruditapes philippinarum and emended description of the genus *Aliivibrio*. Int J Syst Evol Microbiol. 2010;60(1):223–8. 10.1099/ijs.0.010710-0 19648323

[75] Gautier-Bouchardon AV. Antimicrobial resistance in *Mycoplasma* spp. Microbiol Spectr. 2018;6(4):21. 10.1128/microbiolspec.arba-0030-2018 PMC11633602 30003864

[76] Kreizinger Z, Grózner D, Sulyok KM, Nilsson K, Hrivnák V, Bencina D, Gyuranecz M. Antibiotic susceptibility profiles of *Mycoplasma synoviae *strains originating from central and Eastern Europe. BMC Vet Res. 2017;13:10. 10.1186/s12917-017-1266-2 PMC5693497 29149886

[77] Freeman C, Klutman N, Lamp K (1997). Metronidazole–a therapeutic review and update. Drugs.

[78] Farzam K, Quick J (2019). Erythromycin. StatPearls.

[79] Gautier-Bouchardon AV, Reinhardt AK, Kobisch M, Kempf I. In vitro development of resistance to enrofloxacin, erythromycin, tylosin, tiamulin and oxytetracycline in *Mycoplasma gallisepticum*, *Mycoplasma iowae *and *Mycoplasma synoviae*. Vet Microbiol. 2002;88(1):47–58. 10.1016/s0378-1135(02)00087-1 12119137

[80] Hentschel J, Mühlradt PF (1998). Mycoplasma, infection and immunity. Encyclopedia of Immunology.

[81] Schmidt V, Gomez-Chiarri M, Roy C, Smith K, Amaral-Zettler L (2017). Subtle microbiome manipulation using probiotics reduces antibiotic-associated mortality in fish. mSystems.

[82] Pirarat N, Pinpimai K, Endo M, Katagiri T, Ponpornpisit A, Chansue N, Maita M. Modulation of intestinal morphology and immunity in nile tilapia (*Oreochromis niloticus*) by *Lactobacillus rhamnosus* GG. Res Vet Sci. 2011;91(3):e92–7. 10.1016/j.rvsc.2011.02.014 21536310

[83] Ringø E, Hoseinifar SH, Ghosh K, Van Doan H, Becks BR, Song SK. Lactic acid bacteria in finfish-an update. Front Microbiol. 2018;9:37. 10.3389/fmicb.2018.01818 PMC6096003 30147679

[84] Ramírez C, Rojas R, Romero J. Partial evaluation of autochthonous probiotic potential of the gut microbiota of Seriola lalandi. Probiotics Antimicrob Proteins. 2019:1–11. 10.1007/s12602-019-09550-9 31077007

[85] Rawls JF, Mahowald MA, Ley RE, Gordon JI (2006). Reciprocal gut microbiota transplants from zebrafish and mice to germ-free recipients reveal host habitat selection. Cell.

[86] Dang M, Henderson RE, Garraway LA, Zon LI (2016). Long-term drug administration in the adult zebrafish using oral gavage for cancer preclinical studies. Dis Model Mech.

[87] Manichanh C, Reeder J, Gibert P, Varela E, Llopis M, Antolin M, Guigo R, Knight R, Guarner F (2010). Reshaping the gut microbiome with bacterial transplantation and antibiotic intake. Genome Res.

[88] Lozupone CA, Stombaugh JI, Gordon JI, Jansson JK, Knight R (2012). Diversity, stability and resilience of the human gut microbiota. Nature.

[89] Boutin S, Sauvage C, Bernatchez L, Audet C, Derome N (2014). Inter individual variations of the fish skin microbiota: host genetics basis of mutualism?. PLoS One.

[90] Chiarello M, Villéger S, Bouvier C, Bettarel Y, Bouvier T. High diversity of skin-associated bacterial communities of marine fishes is promoted by their high variability among body parts, individuals and species. FEMS Microbiol Ecol. 2015;91(7):12. 10.1093/femsec/fiv061 26048284

[91] Stephens WZ, Burns AR, Stagaman K, Wong S, Rawls JF, Guillemin K, Bohannan BJM (2016). The composition of the zebrafish intestinal microbial community varies across development. ISME J.

[92] Wos-Oxley ML, Bleich A, Oxley AP, Kahl S, Janus LM, Smoczek A, Nahrstedt H, Pils MC, Taudien S, Platzer M, et al. Comparative evaluation of establishing a human gut microbial community within rodent models. Gut Microbes. 2012;3(3):234–49. 10.4161/gmic.19934 PMC3427216 22572831

[93] Ribeiro GO, Oss DB, He Z, Gruninger RJ, Elekwachi C, Forster RJ, Yang W, Beauchemin KA, McAllister TA (2017). Repeated inoculation of cattle rumen with bison rumen contents alters the rumen microbiome and improves nitrogen digestibility in cattle. Sci Rep.

[94] Huang Z, Zeng S, Xiong J, Hou D, Zhou R, Xing C, Wei D, Deng X, Yu L, Wang H (2020). Microecological Koch’s postulates reveal that intestinal microbiota dysbiosis contributes to shrimp white feces syndrome. Microbiome.

