# Supplementary figures and images for: Antibiotic-induced alterations and repopulation dynamics of yellowtail kingfish microbiota
Source: Anim Microbiome. 2020 Aug 3;2:26. doi: 10.1186/s42523-020-00046-4 (PMC7807502; doi:10.1186/s42523-020-00046-4)

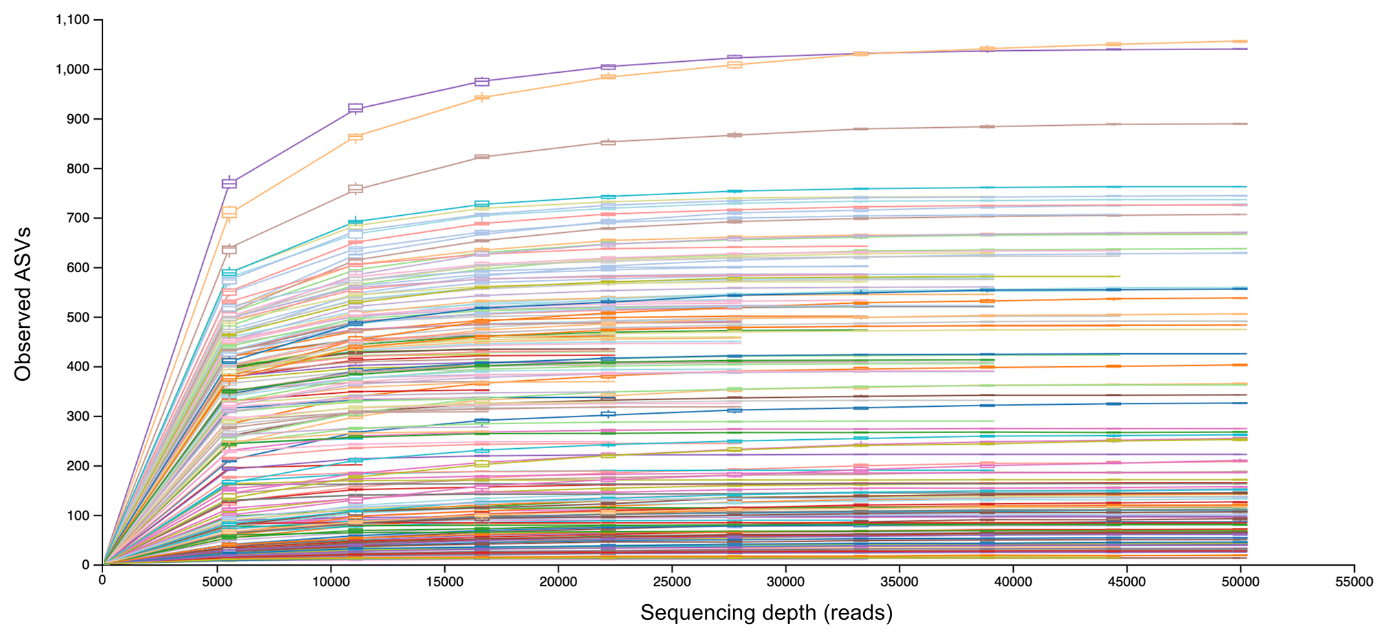


Figure S1


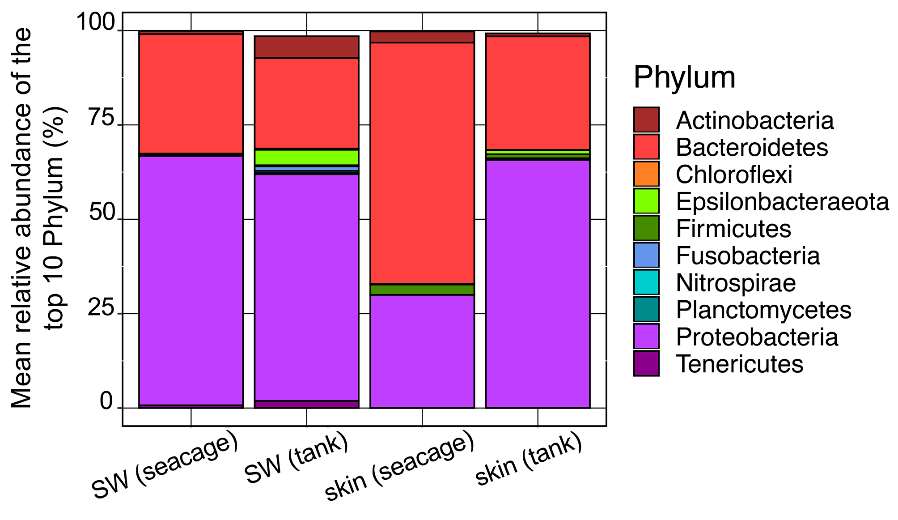


Figure S2


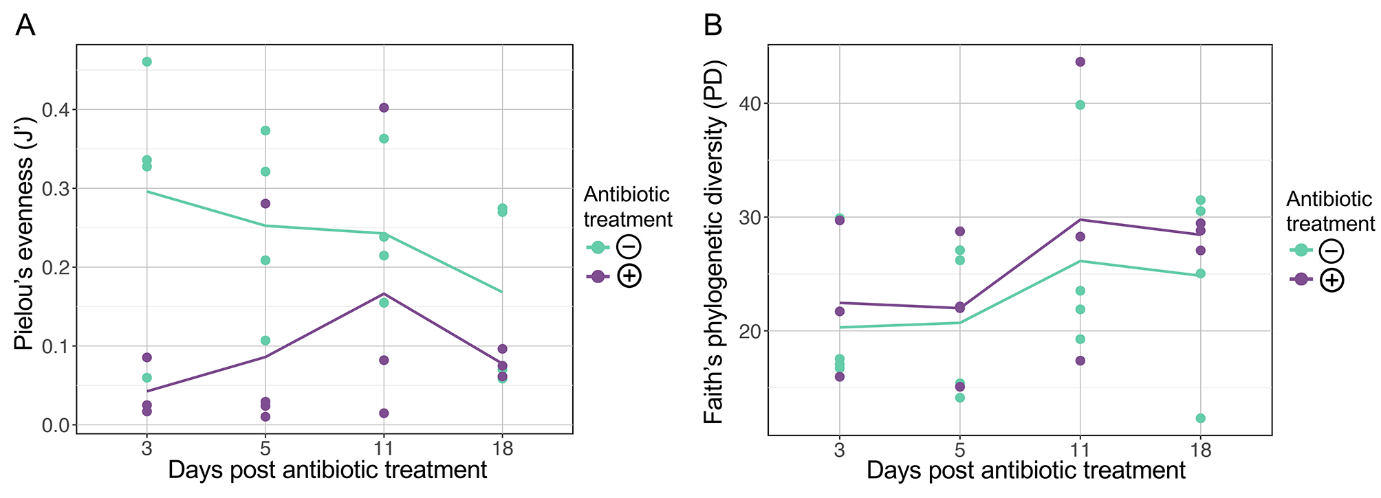


Figure S3


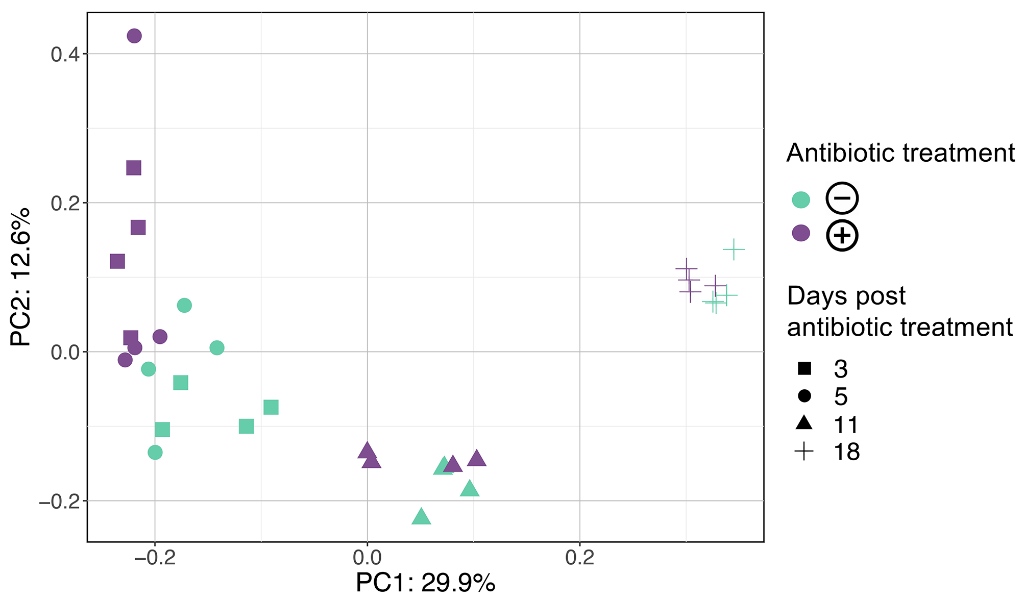


Figure S4


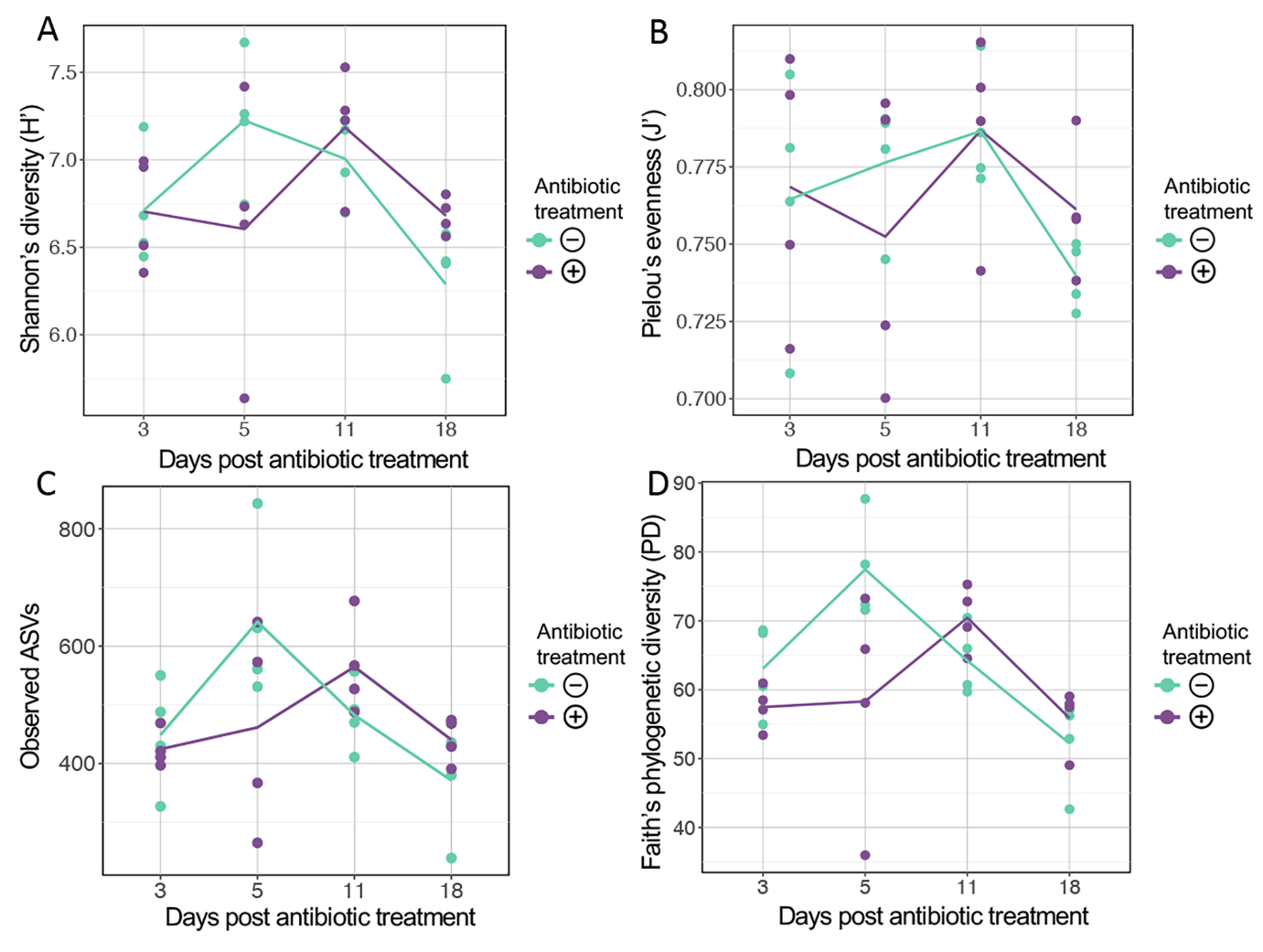


Figure S5


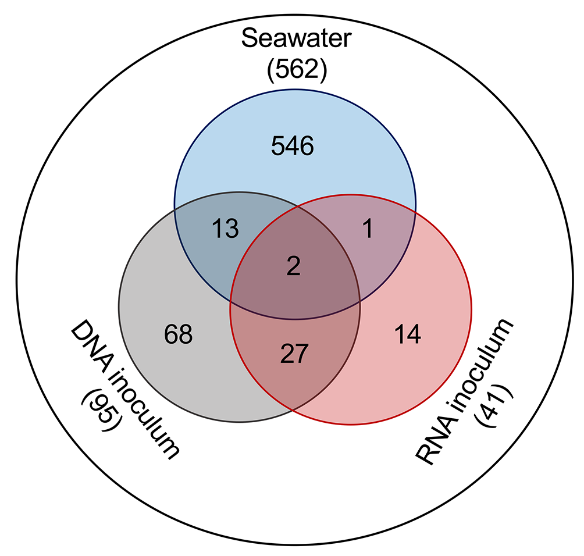


Figure S6


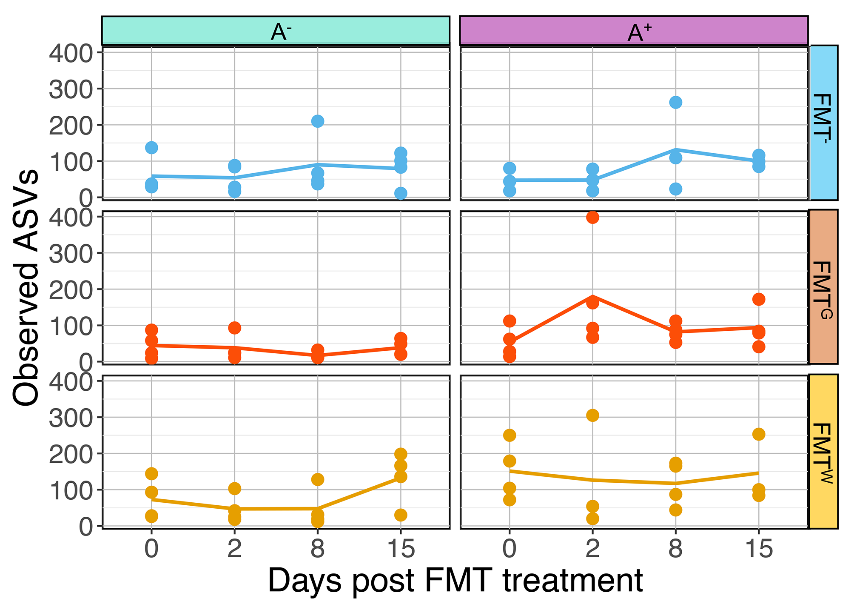


Figure S7

Supplement: Supplementary file 2 — Additional file 2: Figure S1. Rarefaction plot of all samples analysed in this study. Figure S2: Stacked barplots presenting the mean relative abundance (%) of the top 10 bacterial Phylum found in the seawater (SW) and on the skin of fish, comparing fish housed in seacages and those relocated and acclimatised in tanks. Figure S3: Means plots showing the change in mean value of Pielou’s evenness (a) and Faith’s phylogenetic diversity (b) in the gut bacterial communities, over the 18-day treatment period (from n = 4 fish). Figure S4: PCoA plot representing Bray-Curtis similarities comparing the change in global skin bacterial assemblages after treatment with antibiotics (+) over 18-days, with those fish that did not receive treatment (−). Figure S5: Means plots showing the change in mean value of Shannon’s index of diversity (a), Pielou’s evenness (b), total observed ASVs (as a measure of richness) (c) and Faith’s phylogenetic diversity (d) in the skin bacterial communities, over the 18-day treatment period (from n = 4 fish). Figure S6: Venn diagram showing the distribution of unique and shared ASVs in the seawater, and the DNA and RNA inoculum samples. The total number of ASVs within each group are denoted in parentheses. Figure S7: Mean plot presenting the mean number of observed ASVs for the different treatment groups; A−/FMT−, A+/FMT−, A−/FMTG, A+/FMTG, A−/FMTW and A+/FMTW over 15 days post FMT (mean from n = 4 fish). [file 42523_2020_46_MOESM2_ESM.docx]
